# Identifying structural variation in haploid microbial genomes from short-read resequencing data using *breseq*

**DOI:** 10.1186/1471-2164-15-1039

**Published:** 2014-11-29

**Authors:** Jeffrey E Barrick, Geoffrey Colburn, Daniel E Deatherage, Charles C Traverse, Matthew D Strand, Jordan J Borges, David B Knoester, Aaron Reba, Austin G Meyer

**Affiliations:** Department of Molecular Biosciences, Institute for Cellular and Molecular Biology, Center for Systems and Synthetic Biology, Center for Computational Biology and Bioinformatics, The University of Texas at Austin, Austin, TX 78712 USA; Department of Computer Science and Software Engineering, Miami University, Oxford, OH 45056 USA

**Keywords:** Genome resequencing, Experimental evolution, Strain engineering, Insertion sequence, Translocation

## Abstract

**Background:**

Mutations that alter chromosomal structure play critical roles in evolution and disease, including in the origin of new lifestyles and pathogenic traits in microbes. Large-scale rearrangements in genomes are often mediated by recombination events involving new or existing copies of mobile genetic elements, recently duplicated genes, or other repetitive sequences. Most current software programs for predicting structural variation from short-read DNA resequencing data are intended primarily for use on human genomes. They typically disregard information in reads mapping to repeat sequences, and significant post-processing and manual examination of their output is often required to rule out false-positive predictions and precisely describe mutational events.

**Results:**

We have implemented an algorithm for identifying structural variation from DNA resequencing data as part of the *breseq* computational pipeline for predicting mutations in haploid microbial genomes. Our method evaluates the support for new sequence junctions present in a clonal sample from split-read alignments to a reference genome, including matches to repeat sequences. Then, it uses a statistical model of read coverage evenness to accept or reject these predictions. Finally, *breseq* combines predictions of new junctions and deleted chromosomal regions to output biologically relevant descriptions of mutations and their effects on genes. We demonstrate the performance of *breseq* on simulated *Escherichia coli* genomes with deletions generating unique breakpoint sequences, new insertions of mobile genetic elements, and deletions mediated by mobile elements. Then, we reanalyze data from an *E. coli* K-12 mutation accumulation evolution experiment in which structural variation was not previously identified. Transposon insertions and large-scale chromosomal changes detected by *breseq* account for ~25% of spontaneous mutations in this strain. In all cases, we find that *breseq* is able to reliably predict structural variation with modest read-depth coverage of the reference genome (>40-fold).

**Conclusions:**

Using *breseq* to predict structural variation should be useful for studies of microbial epidemiology, experimental evolution, synthetic biology, and genetics when a reference genome for a closely related strain is available. In these cases, *breseq* can discover mutations that may be responsible for important or unintended changes in genomes that might otherwise go undetected.

**Electronic supplementary material:**

The online version of this article (doi:10.1186/1471-2164-15-1039) contains supplementary material, which is available to authorized users.

## Background

Comprehensively identifying genetic variation is critical for understanding the rate and character of genome evolution in laboratory experiments [1], locating the mutations that cause Mendelian diseases, tracking clonal evolution in cancer progression [2], and profiling the emergence of new microbial pathogens and their evolution during chronic infections [3, 4]. Evolutionarily related genomes may exhibit single-nucleotide variation (SNV) and short insertion or deletion (indel) variation, but also structural variation (SV) consisting of larger chromosomal deletions, insertions, and rearrangements. SV may typically have a greater effect on the functions of target genes than SNV and indels, and these types of mutations may also give rise to novel phenotypes that are inaccessible by the other classes of mutations [5]. However, SV is more difficult to predict than SNV and indel variation from DNA sequencing data, particularly short-read data like that produced by many next-generation technologies. Here, we examine the case of genome “resequencing” where a high-quality and complete reference genome exists that is closely related to the DNA sequencing sample that is being analyzed.

Structural variation in genome resequencing data is commonly predicted using two distinct types of evidence derived from how reads map to a reference genome: paired-end mapping (PEM) and split-read alignment (SRA) evidence. Programs such as BreakDancer [6], SVDetect [7], and VariationHunter-CR [8] identify possible SV by examining PEM data for genomic locations associated with read pairs mapped with anomalous insert sizes or pair orientations. A disadvantage of using only this information is that, generally, it cannot resolve the exact location of a breakpoint and the new sequence junction it creates. Thus, programs such as Pindel [9], FusionSeq [10], FusionMap [11], and TopHat2 [12, 13] predict SV using post-processing modules or read-mapping programs that exploit SRA evidence, usually in conjunction with PEM evidence, to determine exact sequence breakpoints. A final strategy for predicting SV is to perform *de novo* assembly of the sequencing reads and then map the resulting contigs back to the reference genome to find discrepancies resulting from large-scale chromosomal changes [14].

Most current software tools for predicting genome structural variation are intended for use with the human genome, or at least a diploid eukaryotic genome. In these large genomes of 100 Mb to 10 Gb, computational speed and memory usage can become an issue, so reads that map to repetitive genomic regions are typically disregarded [6, 7, 9] or may even be removed from a sample prior to sequencing (e.g., by using exome capture). The computational search space for valid SRAs may be further restricted by algorithms that look specifically for gene fusions [10–13] or use species-specific databases of repeats [8]. However, many common types of structural variation can only be detected by exploring read alignments to repeat sequences in genomes, and these existing tools are not easily adapted to haploid microbial genomes without substantial modifications and optimization by the end user.

In lieu of a proven software tool for predicting SVs in microbial genomes, *ad hoc* workflows have been employed by labs that resequence microbial genomes. These approaches may involve manually examining regions where read matches greatly diverge from the reference [15], post-processing the output of SV-prediction tools that examine SRA and PEM evidence [16], or comparing the output of *de novo* assembly tools to the reference genome [17, 18]. Each of these approaches requires significant manual examination of the results to extract true-positives from a high background rate of spurious predictions and to precisely describe the sequence changes caused by mutations that lead to structural variation. This high cost in time and effort means that in many cases, structural variation is simply not analyzed [19, 20]. Thus, only a fraction of the true genetic variation is profiled in many studies of closely related strains.

We have implemented a split-read alignment analysis procedure that accurately predicts mutational events leading to structural variation in clonal samples of haploid microbial genomes as part of the *breseq* computational pipeline [21]. Preliminary versions of the *breseq* SV prediction pipeline described here have been previously used in several experimental studies [5, 22–24]. Here, we fully describe the prediction methods and demonstrate robust performance on simulated *E. coli* resequencing data sets with typical read lengths and depths of genomic coverage. Then, we use *breseq* to identify mutations leading to structural variation that evolved during an evolution experiment with *E. coli* K-12 that was previously used to estimate the spontaneous rates of mutations leading to SNVs and indels in this organism. Mutations leading to SV, in particular transposition of insertion sequences and deletions mediated by these mobile elements, represent ~25% of the spontaneous mutations in this strain. Their prevalence highlights the importance of predicting SV in genetic studies of microorganisms.

## Implementation

### *breseq*overview

*breseq* (pronounced \brēz-ˈsēk\) is an integrated computational pipeline intended for predicting mutations in haploid genomes of less than approximately 20 Mb in total length [21] (Additional file 1). It is implemented in C++ and designed to run as a command-line tool on Unix-like platforms, including Mac OS X and Cygwin. *breseq* requires Samtools (version 0.1.18 is bundled with the code), the read mapper/aligner Bowtie 2 (version 2.1.0 or later; 2.1.0 was used here), and the R statistical computing environment (version 2.14 or later; 2.15.3 was used here). All of these programs are freely downloadable as either source code or compiled executables. *breseq* is invoked using a single command that takes as input FASTQ formatted DNA sequencing reads and reference sequences in GenBank, GFF3, or FASTA format. Providing feature annotations (e.g., locations and names of genes) in the reference files is optional. When present, they are used to optimize the placement of junction breakpoints with respect to mobile elements and to output additional information about the molecular effects of mutations on specific genes.

The *breseq* pipeline uses a reference-sequence-based mapping strategy, which includes evaluating new sequence junctions supported by split-read alignments and tracking multiply-mapped reads, to predict point mutations and structural mutations from short-read DNA resequencing data. As output, *breseq* produces an HTML archive with human-readable summary tables and the relevant evidence for each predicted mutation, including pileups of read alignments and graphs of read-depth coverage. Output is also provided in a machine-readable Genome Diff flat file format that contains entries for predicted mutations (e.g., deletion, base substitution, indel, mobile element insertion) linked to evidence supporting each genome sequence change (e.g., missing sequencing coverage, new sequence junctions, mismatches within aligned reads). The *gdtools* program packaged with *breseq* can be used to manipulate Genome Diff files for further analysis. For example, it can compare mutations predicted in multiple samples or apply changes in a Genome Diff file to generate a mutated version of the genome. Aligned reads and mutation predictions are also output in community formats (e.g., BAM [25] and VCF [26]) that can be directly input into other tools or visualization environments such as Tablet [27] or the Integrative Genomics Viewer [28].

**Figure 1:**
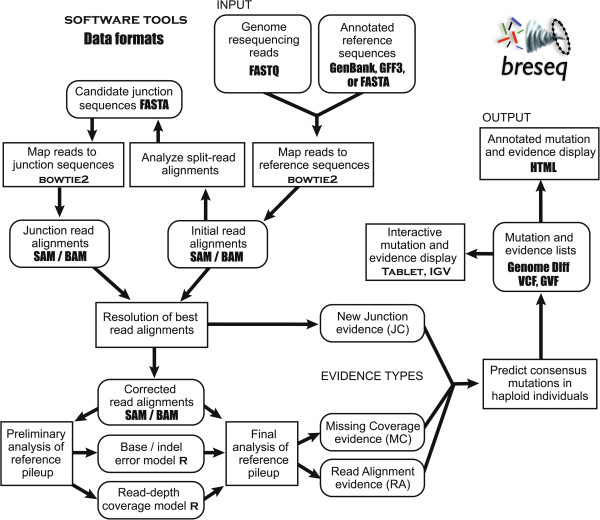
**Overview of the steps used by** ***breseq*** **to identify and annotate mutations in a haploid microbial genome from short-read resequencing data.**

The following sections describe the specific steps of the *breseq* pipeline used to predict new sequence junctions and genomic regions that are missing sequencing coverage in a sample and to infer several types of structural variants from this evidence (Figure 1). Then, we evaluate the performance of *breseq* for detecting these types of mutations in simulated and experimental short-read genome resequencing data sets. The methods that *breseq* uses to predict point mutations and small indels from read alignment evidence are described in the online *breseq* documentation and elsewhere [22, 29], and they are not benchmarked here.

### Mapping reads

*breseq* uses Bowtie2 to map short sequence read data to the reference genome because it performs gapped alignment, finds local matches in the read, and allows exhaustive reporting of all alignments between a read and the reference sequence [30]. Bowtie2 options are used where alignment scores are set to be equal to the number of base matches minus three times the number of base mismatches (down-weighted if they are lower quality base calls), with a gap open penalty of 2 and a gap extension penalty of 3. A staged alignment procedure is employed to accelerate read mapping and alignment. The first, “stringent” phase reports reads with near-perfect matches to the reference genome by using long and sparse seed substrings (seed length of 0.5 times the average read length, constrained to a minimum of 9 and a maximum of 31; seed spacing equal to 1 plus 0.25 times the square root of the read length, rounded down) and a stringent alignment score threshold (0.9 times the read length). Then, remaining unmapped reads are aligned to the reference in a “relaxed” phase with parameters that enable split-read matches to be reported. This stage uses shorter seed substring requirements (seed length of 5 bases plus 0.1 times the average read length, rounded down and constrained to a minimum of 9 and a maximum of 31; seeds spaced by 1 plus 0.25 times the square root of the read length) and a much lower alignment score threshold (6 plus 0.2 times the read length). In both stages, every valid alignment to the reference genome is reported, including all information about how reads map to repeat sequences.

Depending on whether an alignment to the reference genome matches far enough past a large indel for an extended alignment including both sides to achieve a higher score, different reads that overlap the same genomic site may be reported as two separate alignments or a single alignment with gaps. To treat these cases uniformly, *breseq* further splits all initially reported read matches into multiple separate alignments at sites with indels of a certain length or longer (default: 3). In this step, there are cases where, due to inserted bases in the sample that exactly repeat existing reference bases (short duplications), single alignments are rewritten as two overlapping read alignments, rather than simply divided in half, so that the single-alignment mapping results exactly match what would have been output in the two-alignment case.

### Identifying junction candidates

**Figure 2:**
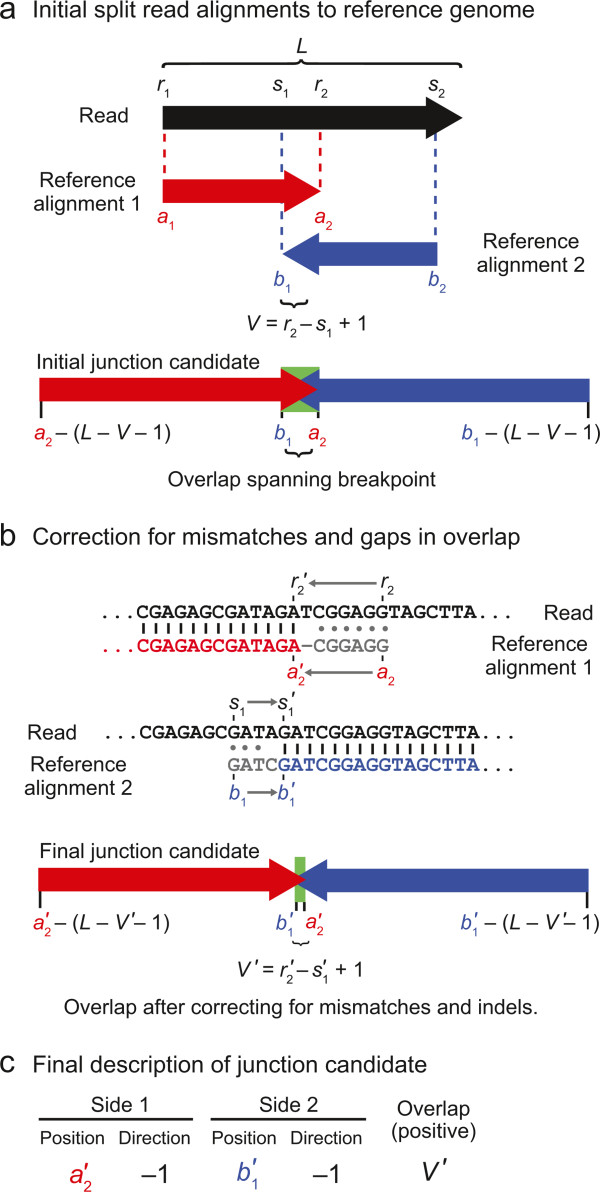
**Junction candidate creation from split-read alignments that overlap. a)** If two alignments of a read to the reference genome overlap, the overlapping bases at the center of the read could potentially be assigned to two separate locations in the reference sequence. **b)** If the read alignments in **(a)** had the imperfect alignments pictured here, the coordinates of each match and their overlap would be corrected as pictured by removing overlap until the remainder is a perfect match with no indels or mismatched bases. **c)** This type of junction candidate can be fully described by the reference coordinates defining each side of the junction breakpoint, the directions in the reference sequence each junction side continues to match from those breakpoint positions, and the number of overlapping bases in the read alignments.

**Figure 3:**
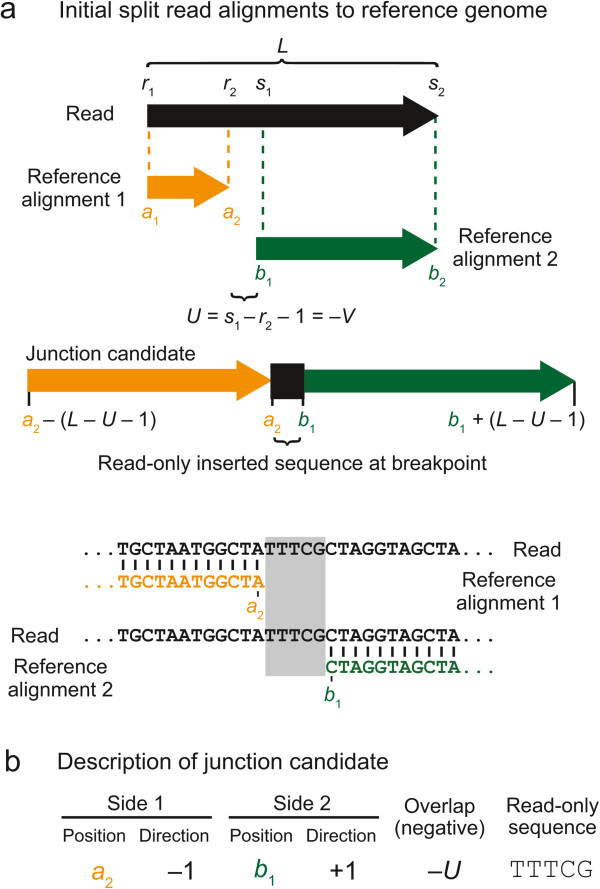
**Junction candidate creation from split-read alignments that do not overlap. a)** If two alignments of a read to the reference genome do not meet or overlap in the middle of the read, then there are unique “read-only” bases present between the two matches to the reference sequence that do not match either side. **b)** This type of junction candidate can be fully described by the reference coordinates on each side of the junction breakpoint, the directions in the reference sequence each junction side continues to match from those positions, and the identity of the read-only bases inserted at the junction breakpoint.

Next, *breseq* creates a list of potential new sequence junctions, which may indicate that distant genomic sites in the reference sequence are juxtaposed in the sample, from the lists of split-read alignments. For all reads where no one alignment spans 90% of the total read length, *breseq* examines all pairs of alignments reported between the read and the reference genome. Each alignment pair uniquely specifies a junction candidate: the new sequence that would exist in the sample DNA if two discontinuous regions of the reference genome were joined (Figures 2 and 3). This approach assumes that cases where individual reads from a sample genuinely map to three or more distinct locations in the reference genome will be extremely rare, which will generally be true for short-read data from samples that have not greatly diverged from the reference genome.

A junction candidate is defined by the endpoints of the first and second matches of the alignment pair to the reference sequence that are nearest the breakpoint (*a*_2_ and *b*_1_) and the directions each sequence extends away from the breakpoint in reference sequence coordinates (whether *a*_2_ > *a*_1_ and whether *b*_2_ > *b*_1_). Additionally, there may be overlap between the two alignments (*V*) because bases at the breakpoint in the read could be assigned to either location in the reference genome without changing the junction sequence (Figure 2a). If there are base mismatches or indels in the initial alignment of this overlap region to either reference location, then each reference alignment is trimmed back until it consists of only perfect matches to the reference genome in the overlap region (Figure 2b). Alternatively, there may be bases at the breakpoint that are unique to the read (*U*). That is, they are not contained within the alignment of either side of the read to the reference genome (Figure 3a). In summary, any junction candidate sequence can be fully specified by six junction description parameters: two reference positions, two orientations with respect to the reference sequence, the number of overlap bases, and the identities of any inserted bases that are in the read and not in the reference (Figures 2c and 3b).

To reduce the number of possible new junction candidates generated, *breseq* only considers pairs of split-read alignments that meet all of the following criteria:

- One alignment must start at the first base of the read (*a*_1_ = *r*_1_).
- If the length of the read is 50 bases or less, the other alignment must end on the final base of the read. For longer reads, the number of unaligned bases at the end of the read must be no more than 10% of the number of bases by which the total read length exceeds 50 bases (if *L* ≤50, then *s*_2_ = *L*; otherwise *s*_2_ ≥ *L* – 0.1 (*L* – 50)).
- The portion of each alignment that does not overlap the other must span a number of bases in the read at least equal to 20% of its total length (min(*s*_1_, *r*_2_) – *r*_1_ + 1 ≥ 0.2 *L* and *s*_2_ – max (*s*_1_, *r*_2_) +1 ≥ 0.2 *L*).
- The number of read bases in the overlap between the alignments must be no more than 12 plus 40% of the number of bases by which the read length exceeds 12 (if *r*_2_ > *s*_1_ + 1, then *V* = *s*_1_ – *r*_2_ – 1 ≤ 12 + 0.4 [*L* – 12]).
- The number of bases unique to the read between the matches to reference sequence must be no more than 12 plus 40% of the number of bases by which the read length exceeds 12 bases (if *s*_1_ > *r*_2_ + 1, then *U* = *s*_1_ – *r*_2_ – 1 ≤ 12 + 0.4 [*L* – 12]).
- Only alignment pairs with the maximum value of the number of bases spanned in the read by the two alignments (*s*_2_ – *r*_1_ + 1) that is observed over all alignment pairs for a given read are eligible to construct junction candidates.

For all alignment pairs for a read that pass these guards, a junction candidate sequence is constructed by taking the corresponding regions of the reference genome and joining them together, accounting for any overlap between the alignments or for any intermediate bases that are in the read but not in the reference genome. The junction candidate sequence is extended on each end by adding enough bases to ensure that if the longest read in the input data set mapped to the junction across its breakpoint it would have a better alignment score than the best match to anywhere in the reference genome. This step requires adding a number of bases from the reference to each side equal to one fewer than the maximum read length in the sample, minus the overlap (*V*) or read-only sequence (*U*) size.

While processing all reads with suitable split mappings to the reference sequence in this manner, the lists of predicted junctions with the exact same junction sequence and junction description parameters are merged into a running list of possibilities. So that two equivalent junction candidates will be merged together regardless of which DNA strand the original reads matched, junction sequences are reverse complemented, if necessary, so that the first side (left in the orientation of the junction sequence) is always described by the first reference sequence matched of those provided as input (in alphabetical sort order by sequence ID) or by the lowest reference coordinate matched if both sides align to the same reference sequence.

Because *breseq* uses read mapping options that report all alignments to sequence repeats in the reference genome, pairs of alignments for the same read that match different reference locations may yield equivalent junction sequences but different junction descriptions. For example, reads that map to the boundary of a new insertion of a mobile element that is multicopy in the original genome will have multiple alignments to the reference sequence for the portion overlapping the mobile element. Due to differences in the flanking bases at the boundaries of different copies of the multicopy repeat sequence, the same junction may also be described with a different number of overlapping bases or inserted read-only bases at the breakpoint for each of these possibilities. Therefore, *breseq* next collapses the list of junction predictions further: to the set of the shortest junction candidate sequences that are subsequences of other sequences or their reverse complements, effectively favoring those junctions with the least overlap or smallest number of unique read bases. In this merging step, it also prefers to keep junctions in which both ends map to the same reference sequence fragment (e.g., chromosome), rather than different ones, and in which the two ends match nearby coordinates if they are on the same reference sequence.

**Figure 4:**
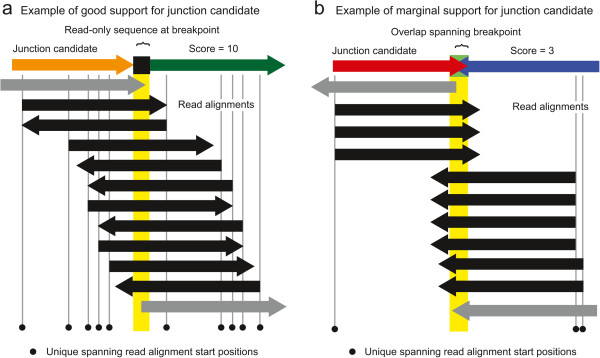
**Example of assigning coverage evenness scores to candidate junctions.** Reads that align to a candidate new junction sequence may start at many different positions relative to the breakpoint. Reads that do not unambiguously support the new junction (gray arrows) because they do not extend across the breakpoint and any overlap or read-only bases (yellow highlighting) are not counted toward the evenness score. Although the two examples have the same number of reads that support the new junction because they align across the breakpoint and match the junction better than the reference genome (black arrows), the example in **(a)** is well-supported because these reads start in many different registers with respect to the breakpoint as would be expected for a normal reference genome location, whereas the example in **(b)** has reads beginning at a small number of biased positions with respect to the junction. This coverage evenness score is used to calculate a skew *p*-value to accept or reject a candidate junction, after also accounting for differences in the maximum number of read start positions that can support each candidate junction. In cases of tandem duplications much shorter than the read length, reads must also extend several “continuation” bases past any unique-only or overlap sequence to count as supporting a junction, as illustrated in Figure 5.

**Figure 5:**
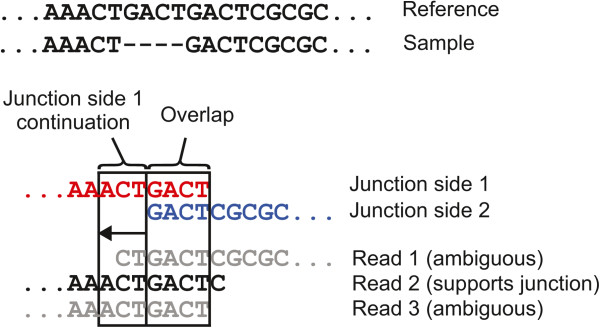
**Case where additional read continuation across a breakpoint is required to support a junction candidate.** In certain cases a read alignment must extend further across a junction breakpoint than just through the alignment overlap or read-only sequences to support the junction versus aligning equally well to the original reference sequence. One such case, where there is a deletion of four bases in a short tandem repeat region is shown. In this example, read alignments to the junction candidate sequence must extend across the four overlapping junction bases and the three bases shown on their left side to support the junction.

To determine the best merged junction candidates to further test, *breseq* next assigns a coverage evenness score to each one (Figure 4). This score is equal to the number of distinct start positions for alignments of reads that extend across the breakpoint far enough to unambiguously support the junction and not the reference sequence. That is, they must span any overlap or read-only bases in the junction sequence. If the junction is a short deletion of a few bases in the reference sequence, then it may be required to extend additional bases that are not accounted for in these values — a continuation length — in order to unambiguously support the junction and count toward the actual or possible coverage evenness score (Figure 5). Each read is counted as starting at the position in the reference genome where its first base matches, so alignments with the same start and end coordinates, but on opposite strands will each count toward this evenness score once.

Reads overlapping a typical position in the reference genome will have start sites that are relatively evenly distributed before and after that position for reads that align to the top and bottom strands, respectively (Figure 4a). New junctions that are the result of mutations should similarly be supported by reads that cross the breakpoint in many different registers. In contrast, junction candidates resulting from sequencing artifacts tend to be supported by reads that align unevenly across a junction and may have low evenness scores even when they have very high coverage (Figure 4b). This situation can result from reads with systematic errors, like homopolymeric base calls at their ends, or from reads in the sample that are so significantly different from the reference sequence that they are no longer accurately mapped.

Finally, the list of junction candidates is sorted from high to low by this coverage evenness score and only the top candidates are saved according to an iterative decision procedure. At each step, all junctions with the next-highest score in the list are considered for inclusion. If the new total number of junction candidates accepted to this point, including these, would exceed some maximum (default: 5000) or the cumulative length of junction candidate sequences to this point would exceed a threshold value (default: 10% of the reference genome length), then these and all lower-scoring junctions are not retained, as long as some minimum number of junction candidates has already been accepted at this point (default: 100). In addition to these criteria, a junction candidate must have at least some minimum evenness score to be considered (default: 2).

### Evaluating new junction evidence

Some sequencing reads may map reasonably well to the original reference genome but actually align better to a junction candidate sequence. This situation can arise, for example, when the junction sequence spans an insertion or deletion of several bases in the sample relative to the reference. For junctions between sequences that were distant in the original reference genome, this situation can also occur when a read is not initially mappable to both sides of a junction because it does not extend far enough past the breakpoint to seed and detect a match to one side or the other. To resolve and properly count these cases when evaluating junctions, *breseq* re-maps all input reads to the set of candidate junction sequences using Bowtie2 with the stringent criteria for reporting alignments that were used in the first stage of mapping to the reference genome. For each read, *breseq* compares the alignment scores of the best matches to junction sequences and the reference sequence. If a read matches the reference genome better than all junction candidates, then it is immediately assigned to that location and not considered further with respect to junctions. If it matches a junction or multiple junctions better than or equally as well as the reference genome, then the read is temporarily saved with those junctions in an unresolved state until evidence from all reads has been compiled.

To evaluate whether to call a genomic variant in the sample, one would like to determine whether the final collection of reads that align to a candidate junction resembles those found at a typical site in the reference genome. To this end, the coverage evenness score — equal to the number of unique reference positions matched by the first bases of reads extending past the breakpoint and any overlap or read-only junction bases (Figure 4) — is recalculated based on the re-aligned reads that match best to each junction. This score is now used to rule out junction candidates with unusually low coverage or coverage biased toward certain registers with respect to the breakpoint that may be the result of mapping artifacts, contaminating sequences, or failed reads. This general approach has been used to manually screen out false-positive predictions of gene fusions [31] and incorporated into the scoring scheme used by TopHat2 [12]. We score the evenness of read coverage across a predicted breakpoint to evaluate the support for a new sequence junction in the context of a statistical model of read coverage across a haploid genome.

**Figure 6:**
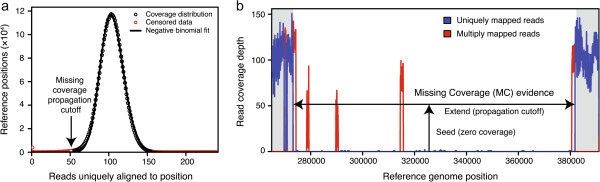
**Missing coverage evidence. a)** The censored fit of read depth at sites with unique-only coverage across the reference genome to a negative binomial distribution is shown for one of the *E. coli* samples from the mutation accumulation evolution experiment. The threshold for extending putative deleted regions of the genome is determined by taking the coverage value that produces a left-tail probability from the fit distribution as described in the text (arrow). **b)** A missing coverage evidence item is shown for the same *E. coli* sample to illustrate how its boundaries are determined by extending outward from a seed region with zero coverage of uniquely aligned reads through regions with multiply-mapped reads that match genomic repeat sequences until the coverage of uniquely aligned reads exceeds the calculated propagation threshold. Note that the left and right boundaries both correspond to a range of positions because they fall within repeat regions. In some cases, this type of ambiguity in the extent of the deletion can be resolved by examining new junction evidence matching the endpoints.

First, to calibrate whether any given coverage evenness score is unusually low with respect to the expectation for a typical position in the reference genome, *breseq* fits the distribution of coverage read depth across the genome and determines the average chance that at least one read starts at a given position of the reference sequence. These parameters are estimated from the initial best mappings of reads to the reference genome before resolving candidate junctions. For short-read resequencing data, the depth of read coverage at different positions in the reference genome is fit well by an overdispersed Poisson (negative binomial) distribution [29]. *breseq* fits this distribution using unique-only reference genome positions (those not matched by any read that maps to multiple reference locations equally well) (Figure 6a). Before fitting, this data is left-censored at half the average coverage, to account for positions that are truly deleted in the sample but may have a small amount of residual coverage due to incorrect mapping of reads with errors or cross-contamination from sequencing similar genome samples without the deletion at the same time. The data is also right-censored at 1.5 times the average coverage so that fitting will be more robust against cases where failed sequencing reads may spuriously map to a small number of genomic locations, creating anomalously high coverage, and to cases where there are increases in copy-number in a sample relative to the reference across a significant portion of the genome. The negative binomial distribution is described by the mean coverage (*μ*_*cov*_) and a size parameter (*α*_*cov*_) reflecting the overdispersion. As coverage is tabulated, the number of unique-only positions with no reads beginning there that match a given strand (forward or reverse) is also tracked for each reference sequence. Dividing this total count by twice the number of unique-only positions in a reference sequence gives , the average chance that no read will be found to start at a given position extending across a breakpoint, i.e., the chance that a possible position where a read could have started will contribute to the evenness score for a junction.

After creating this model of the statistics of the evenness score, *breseq* iterates through all junction candidates, beginning with the one with the highest coverage evenness score, and calculates how extreme this score is relative to expectations to accept or reject the prediction. In cases where reads are currently unresolved because they matched equally well to more than one junction sequence, these reads are temporarily assigned to the current junction and included in its recalculated evenness score at this point. The possible values that the coverage evenness score can take for a junction are determined by the maximum read length, but when reads with different lengths are present in a data set, the average read length  gives a better idea of what values of the evenness score to expect. In addition, junctions with overlap bases or unique read-only bases will have fewer possibilities for reads to be positioned such that they align better to the junction sequence than to the reference genome, and therefore contribute to the coverage evenness score (Figure 4). As described above, it is also possible – particularly for deletions or duplications of short sequence repeats – that a read aligning from one side of the junction would not be distinguishable as matching the junction better than the reference unless it aligned a certain number of additional bases past the breakpoint (Figure 5). To account for both of these considerations, the expected maximum possible evenness score (*S*_*max*_) for a particular junction candidate is revised downward by taking into account the overlap (*a*_2_ − *b*_1_ + 1) and the numbers of ambiguous continuation bases on side one (*C*_1_) and side two (*C*_2_):
1

The chance of observing a given evenness score at a typical reference position is calculated by integrating the chances that a certain depth of read coverage would occur and the chance that one would observe the given evenness score at that read depth given the chance that a position would not have any reads with alignments starting at that position on a given strand  and would, thus, not contribute to the score. Therefore, *breseq* calculates a *p*-value for rejecting the null hypothesis that the start position and strand distribution of reads across each junction is typical of the rest of the genome according to the following equation:
2

where *NegBinom* (*x*, *μ*, *α*) is the probability of observing *x* events from the negative binomial distribution with mean *μ* and size parameter *α*, and *BinomCDF* (*x*, *p*, *n*) is the chance of *x* of fewer successes in *n* draws with probability *p* of a success each time. The probability of success in this case is the chance that a read will be observed beginning at a given position and on a given strand at an arbitrary position in the reference genome as a whole, estimated as described above. This calculation uses a value for this chance that scales with the current coverage in the summation relative to the average coverage of the reference sequence. If the two sides of the junction match two different reference sequences (which may have different coverage-evenness properties), then a *p*-value is calculated for each one and the junction is assigned the least-significant value.

The “evenness skew” for the junction candidate is defined as the negative base-10 logarithm of this *p*-value, and *breseq* rejects predictions with skew scores that exceed some threshold (default: 3.0). For accepted junctions, any unresolved read alignments that mapped equally well to other junctions are claimed by the accepted junction prediction and are not counted toward the score of alternative junctions they matched equally well when they are considered later. The top-scoring rejected junctions are saved for reporting as marginal predictions in the output.

For accepted junctions, ambiguous placement of the breakpoint with respect to the two disjoint locations of matches in the reference sequence due to overlap is assigned to one side or the other according to the following rules. First, if reference sequence annotations of type “repeat sequence” or “mobile element” are present, and the breakpoint is within a certain distance of the end of one of these features (default: 20 bases), then the breakpoint is shifted to exactly align to the end of the annotated feature. This may involve assigning all of the overlap bases near the breakpoint to one side, or some of these bases to both sides of the junction. Second, if one side of the junction was marked as having only repeat sequence alignments during the phase when candidate junction sequences were constructed, then all of the ambiguous bases at the breakpoint are assigned to the side that had only unique alignments. If both sides of the junction had unique alignments, then the side with the highest priority when sorting by reference sequence ID and then by coordinate, is assigned all overlap bases. Reads mapping across successful junctions are split at the breakpoint and the two pieces are added to the final file of alignments to the reference genome as separate matches so that they are counted correctly toward predictions of other mutations, such as base substitutions.

### Evaluating missing coverage evidence

In addition to compiling new junction evidence for structural variants as detailed above, *breseq* includes a step where it examines read coverage for evidence of deleted chromosomal regions. To do this, it first identifies reference positions where there is zero coverage of both uniquely-mapped and multiply-mapped reads. These seed intervals are propagated outward and joined through areas where the total of the unique-only read coverage is below a cutoff value. This threshold is calculated from the negative binomial fit to the coverage distribution (discussed above) by choosing a coverage value that yields a left-tail probability of 0.05 divided by the square root of the length of the current reference sequence (Figure 6a). Note that this procedure may lead to extending and joining putative missing coverage intervals through regions where multiply-mapped reads align (Figure 6b). When these occur between areas where there is unique-only coverage below a certain threshold or no coverage at all, it is assumed that those regions are also part of a deletion because the reads mapping there match equally well to a region elsewhere in the genome that was not deleted. When a region with multiply-mapped reads occurs on the margin of a region of missing coverage, the endpoint of the predicted deletion on that side is considered ambiguous, unless resolved by integrating this evidence with junction predictions as described below.

### Mutation annotation

**Figure 7:**
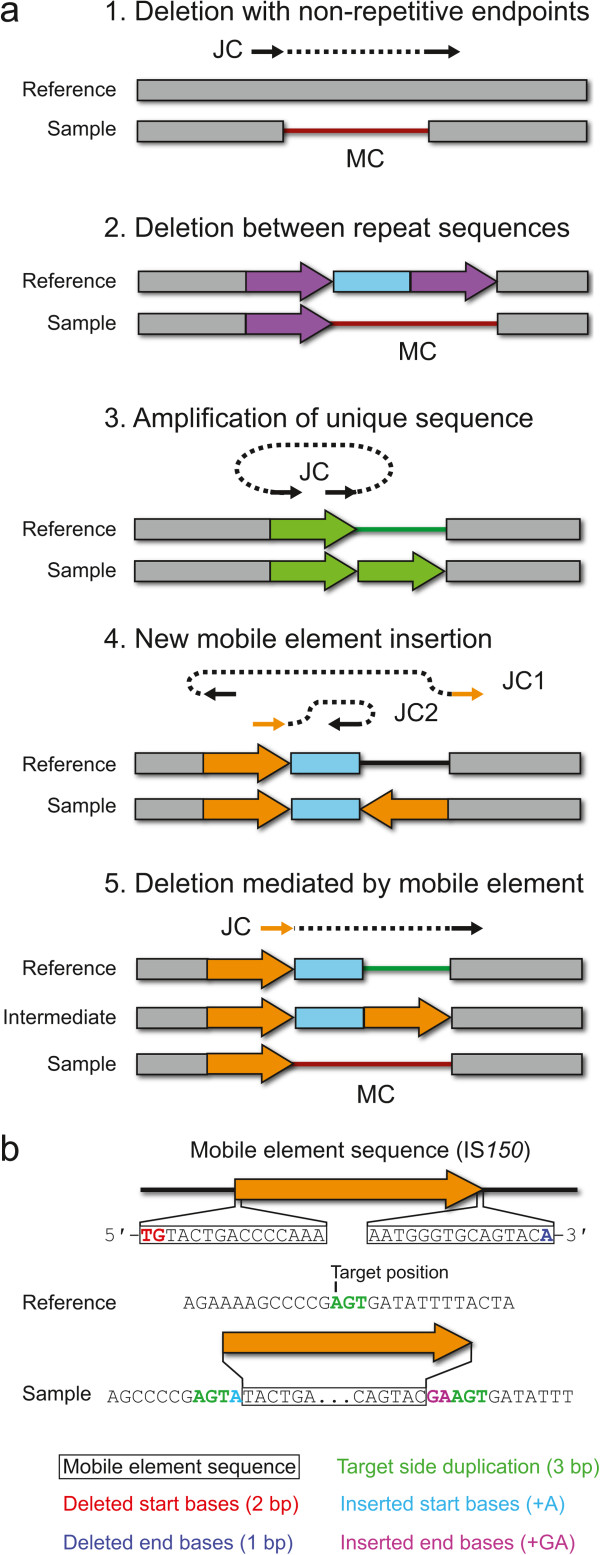
**Predicting structural variation from new junction and missing coverage evidence. a)** Types of structural variation for which *breseq* can predict precise mutational events from new junction sequences (JC) and missing read coverage (MC) evidence are shown in the context of the reference and mutant genomes. For JC evidence, the matched sequence on each side is shown as a solid arrow with a dashed line connecting the two sides. Orange JC arrows indicate that this side of a new sequence junction maps equally well to multiple locations in the reference genome (i.e., the location is ambiguous). Details for the procedure used in each case are described in the text. **b)** Mobile element insertions may require additional fields to describe the precise sequence change caused by insertion of a new copy. These may include a target site duplication and deleted or inserted bases on the margins of the new element copy, as shown.

*breseq* predicts and annotates several types of mutations that produce structural variation by considering new junction (JC) and missing coverage (MC) evidence together with reference sequence annotations of the locations of mobile elements (Figure 7a). This automated integration of information results in precise (i.e., down to the exact nucleotide) and biologically meaningful (e.g., insertion of a new copy of the transposable element IS*150* with duplication of three target site base pairs) predictions of how a sequence is altered in the sample relative to the reference that would be laborious to reconstruct from the output of other programs that could potentially be used to predict SV in microbial genomes. The predictions by *breseq* include:

1. *Large deletions creating unique junctions* (JC + MC evidence) – When the endpoints of a missing coverage evidence item exactly match the breakpoint in a junction evidence item with two uniquely aligned sides, the region between them is predicted as a deletion. Deletions of this kind can be caused by illegitimate recombination.
2. *Deletions between by two copies of a repeat element* (MC evidence) – When the ends of a missing coverage evidence item both fall within regions of multiply-mapped read coverage that are annotated in the reference genome as copies of the same repeat element in the same orientation, then a deletion is predicted that includes the intervening region between the repeats and the second of the two repeat copies. These types of deletions may occur via homologous recombination.
3. *Short deletions, insertions, substitutions, and tandem amplifications* (JC evidence) – When the two sides of a new junction evidence item are uniquely aligned and the breakpoint positions are located within a distance of each other in the reference genome that is smaller than the average read length, then they are resolved to predict the corresponding change. Note that deletions are predicted even without missing coverage evidence in this case because it is possible for one or a few spurious reads or read alignments to prevent the prediction of a very small missing coverage evidence item.
4. *New mobile element insertions* (JC + JC evidence) – When two junction evidence items have unique endpoints on one side that are located within 20 base pairs of one another and match opposite sides of the same repeat element family annotated in the reference genome, then they are resolved to predict a new insertion of a mobile element. A target site duplication of several bases typically occurs when a new copy of a bacterial transposable element inserts into a genome. The size of this duplication is predicted from the locations of the junctions. Additional deletions of a few bases from the ends of the newly inserted element relative to its consensus sequence or insertions of a few bases within the target site duplication may be necessary to reconcile the exact breakpoint sequences in some cases (Figure 7b).
5. *Deletions mediated by mobile elements* (JC + MC evidence) – These mutations are predicted when a missing coverage evidence item exists with one side overlapping a repeat sequence in the reference genome and a new junction evidence item matches the unique side of the missing coverage interval and the proximal side of any copy of the corresponding repeat family in the genome. In this case, a deletion is predicted that extends from the unique breakpoint on one side of the repeat element up to its boundary.

Missing coverage and accepted new junctions that are not resolved into predictions of precise mutational events are displayed as “unassigned evidence” in the HTML output for further evaluation by the user. For example, *breseq* currently does not attempt to resolve new junction evidence that could support tandem sequence duplications greater than the read length. It also does not attempt to use new junction evidence items in which both sides fall in repeated reference genome sequences to predict mutations. These cases and other more complex types of events can potentially be resolved by manually examining unassigned evidence items, read-depth coverage graphs, and the relative locations of repeated sequences in the genome to predict the most likely mutational event [21]. In addition, the top-scoring junctions that were rejected according to the statistical tests described above are listed on a separate page that contains “marginal evidence” to potentially aid in resolving other unassigned evidence items.

## Results and discussion

### Simulated data sets

To evaluate the performance of junction prediction by *breseq* we first computationally mutated the *E. coli* B REL606 genome sequence [32]. Each simulated genome had many instances of a single type of structural variation that can be detected because it creates new sequence junctions: (1) *Large deletions creating unique junctions*. For each test genome, 100 deletions of 400–1000 base pairs that created junctions between non-repeat sequences were simulated. (2) *New mobile element insertions.* An insertion sequence (IS) is a bacterial transposable element that is capable of inserting a new copy of its sequence elsewhere in the genome [33]. For each test genome, 100 new IS insertions were simulated with the newly inserted sequence randomly chosen from existing IS element copies. The mechanism of IS integration typically results in a short duplication of a few bases adjacent to the target site (Figure 7b). We randomly chose to duplicate 1–10 bases for each simulated insertion. (3) *Deletions mediated by mobile elements*. DNA cleavage or insertion of a new IS copy may lead to a deletion flanking a nearby, existing copy of an identical IS element in a reference genome with the same orientation [34], which eliminates the intervening sequence and one IS copy. For each test genome, only 40 of these events were simulated because there are only 49 total annotated IS elements in the reference genome that could be chosen to mediate an adjacent deletion. We selected these three types of mutations because they are commonly found in laboratory evolution experiments with this strain [22].

To prevent the endpoints of these mutations from falling in repeat sequences that would lead to ambiguity in predicting their locations from short-read resequencing data and from occurring adjacent to one another and leading to more complicated genetic changes, we further restricted the possible locations for each simulated mutation as follows. First, we enumerated all exact sequence repeats of ≥36 bases in the reference genome using MUMmer (version 3.23) [35]. Then, we required that the sites affected by each mutation be spaced at least 1,000 bases from one another and from every one of these exact sequence repeats. For generating IS-mediated deletions, an existing IS element was randomly chosen and the deletion extended from either the left or right margin with equal probability. Another deletion mediated by the same IS element on its other side, was ruled out in adding further mutations to that simulated genome.

We created enough test genomes to have a total of 1,000 mutations in each of the three categories. The Mason read simulator (version 0.1.2) was used to simulate FASTQ files of next-generation sequencing reads that would be obtained by sequencing these reference genomes [36]. Specifically, we generated fragment (single-end) read data sets under the Illumina model with exact read lengths of 36, 50, 100, and 200 bases; and under the Roche 454 model with varying read lengths averaging 100, 200, and 400 bases with a standard deviation of 10%. To test software performance with different levels of read coverage depth, we simulated FASTQ files with 10-, 20-, 40- and 80-fold genomic coverage for each technology and read length.

The simulated data sets were analyzed by *breseq* (version 0.25) using default settings. The same junction sequence can potentially be described in many different yet equivalent ways using our junction description scheme: the breakpoint coordinates can be shifted when there is overlap between the two read matches, or different coordinates and strands can be used to describe a side of a junction that matches a repeated sequence in the reference genome. Therefore, we compared the *breseq* predictions to our input structural variants at the level of junction sequences rather than junction descriptions. For the *breseq* predictions and the original file of input variants, we generated the corresponding junction sequences from the reference genome and added five fewer bases than the average read length being tested as flanking bases on each end of the overlap region at the breakpoint. If the sequence of a junction in the *breseq* predictions matched the sequence of a junction in the simulated genome or one of the two junction sequences was an exact subsequence of the other, they were judged to be equivalent predictions.

**Figure 8:**
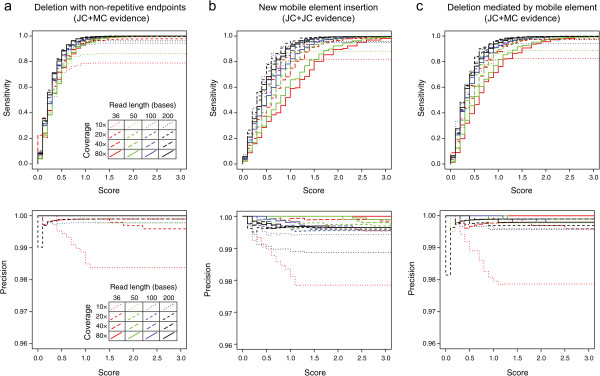
**Performance of structural variant prediction on simulated Illumina data sets.** Data sets with different read lengths and coverage depths were generated according to an Illumina error model from simulated *E. coli* reference sequences with many examples of a single type of mutation causing structural variation randomly introduced. Results in terms of the sensitivity (or recall) for recovering true-positives (top panels) and the precision, equal to the number of true-positive predictions over the total number of predictions (bottom panels), are graphed as a function of junction skew scores accepted for making predictions. Results are shown for simulated genomes containing only **a)** deletions with breakpoints in non-repetitive reference genome sequences, **b)** new insertions of bacterial transposable sequences (IS elements), and **c)** deletions with one boundary ending on a repetitive IS element. The default junction skew score cutoff used by *breseq* is 3.0.

**Figure 9:**
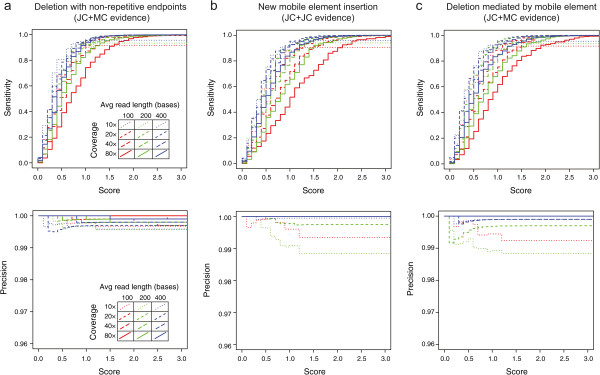
**Performance of structural variant prediction on simulated 454 data sets.** Read data sets with different average read lengths and coverage depths were generated according to a 454 error model with a 10% standard deviation in read length from simulated *E. coli* reference sequences with many examples of a single type of mutation causing structural variation randomly introduced. Results in terms of the sensitivity (or recall) for recovering true-positives (top panels) and the precision, equal to the number of true-positive predictions over the total number of predictions (bottom panels), are graphed as a function of junction skew scores accepted for making predictions. Results are shown for simulated genomes containing only **a)** deletions with breakpoints in non-repetitive reference genome sequences, **b)** new insertions of bacterial transposable sequences (IS elements), and **c)** deletions with one boundary ending on a repetitive IS element. The default junction skew score cutoff used by *breseq* is 3.0.

We evaluated the sensitivity (fraction of true-positive junctions predicted) and precision (number of true-positive predictions divided by the total number of junction predictions) for each simulated data set (Figures 8 and 9). All analyses used the default parameters except for the simulated 400-base 454 reads with the new mobile element insertions, for which the maximum cumulative length of junction candidate sequences was increased to 25% of the reference genome length and the minimum number of candidate junctions tested was increased to 250 so that enough of the longer junction candidates were tested to enable evaluation of all 200 true-positive junctions. Predictions of these mutations by *breseq* were highly specific and sensitive for all technologies and read lengths when there was at least 20-fold coverage of the reference genome. These results show that the skew score provides a good statistical cutoff at the default significance level (*p* = 0.001) for predicting the new sequence junctions created by all three categories of mutations.

We next tested the prediction of junctions in simulated data sets with very high read-depth coverage to determine whether the evenness skew score would become saturated, such that it no longer provided an effective metric for evaluating junctions. This situation might occur when reads exist that match the reference starting at nearly every reference position on both strands. We used Mason to generate a series of Illumina single-end 50-base read data sets for one of the simulated genomes with 100 deletions. These data sets progressively included additional reads to achieve 80-, 160-, 320-, 640-, 1,280-, and 2,560-fold genomic coverage. For each of these samples, *breseq* identified the 100 junctions present in simulated genome perfectly (all true-positive predictions). At the higher coverage values we observed more spurious junction candidates, but these were all rejected on the basis of their skew scores.

### Reanalysis of an *E. coli*evolution experiment

Because the simulated data does not fully reflect the types of sequencing errors present in a real data set, we next used *breseq* to predict mutations in genome resequencing data from a mutation accumulation (MA) laboratory evolution experiment with *E. coli* [19]. In bacterial MA experiments, replicate lineages are cultured for many generations by picking an arbitrary colony and streaking it out on a new agar plate each day. Due to the random choice of which individual colony is chosen and the frequent bottlenecks each time a subsequent colony grows from a single cell, this procedure leads to mutations accumulating over time in a lineage with very little influence from their fitness effects on the organism, as long as they are not lethal. Therefore, the number of mutations that accumulate over time in a MA experiment can be used to estimate the rate of spontaneous mutations in an organism [1].

Point mutations and short indels (≤4 base pairs) were previously identified in the genomes of *E. coli* clones isolated at the end of this MA experiment, but structural variation was not analyzed. We tested SV prediction by *breseq* with default settings on the 21 genomes that had evolved for 6,000 generations as part of this MA experiment. The Illumina resequencing data sets for these genomes consist of paired-end data with 90-base reads and an average coverage depth of approximately 100-fold. We first examined *breseq* output for genetic differences from the *E. coli* K-12 MG1655 (GenBank: NC_000913.2) reference genome that were shared by all 21 MA clones. These mutations were likely present in the MA experiment ancestor, so we generated an updated ancestral genome sequence incorporating these changes and used this as input into *breseq* to predict new mutations that evolved independently in each of the 21 MA lineages (Additional file 2).

In total, *breseq* predicted 48 evolved mutations that led to structural variation — 8 deletions, 3 IS-mediated deletions, 2 tandem duplications, and 35 IS-element insertions — from 84 new junction and 9 missing coverage evidence items. All of the remaining 5 unassigned new junction evidence items and 1 missing coverage item could be manually resolved into 4 additional mutations: 2 IS-element insertions located in genomic repeat sequences that made one or both of the supporting junctions match multiple sites in the reference sequence equally well; 1 deletion between IS elements in which at least one of the two supporting junctions had one side that was ambiguously placed; and 1 tandem repeat of 100 bases that was not automatically predicted because this is longer than the read length.

Manually examining all evidence items also showed that one prediction of a point mutation was spurious and actually resulted from the deletion of one 8-bp unit from a sequence repeat that initially consisted of eight of these 8-bp units in the reference genome. Several pieces of unassigned missing coverage evidence (3 to 17 per genome) appeared to be due to locally low read coverage that dipped to zero at locations in the genome. As many of the chromosomal regions with this missing coverage were found in common between multiple independently evolved MA lines, it is possible that they result from systematic biases in coverage due to a library preparation artifact or more complex mutations that are already present in the ancestral strain of *E. coli* but cannot be resolved from mapping short-read data to the reference sequence.

New junctions were also predicted that correspond to a 1829-bp inversion caused by the *pinE* recombinase within the e14 prophage [37]. The ancestral *E. coli* strain appeared to have the P(−) orientation instead of the P(+) orientation in the MG1655 reference sequence. Reversions to the P(+) orientation were found in some of the evolved genomes. In most cases these mutations were not present in 100% of the sample, as determined by counting the number of reads aligned to the P(−) form versus the P(+) form in the same data set, perhaps indicating that this region may be genetically unstable and commonly “switch” during outgrowth of the selected clone, creating a mixture before genomic DNA is isolated for sequencing.

In addition to these mutations giving rise to SV, *breseq* predicted 19 indels (≤4 base pairs) and 154 base substitutions in these genomes. These values are slightly higher in each case than the 12 and 140, respectively, predicted in the initial report [19]. This discrepancy is likely due to differences in the stringency of read alignment and quality control cutoffs, as all of the *breseq* indel and SNV predictions appeared to be high quality upon manual examination. We cannot directly evaluate the performance of *breseq* on this *E. coli* data since the full set of true-positive mutations is unknown. However, we note that in other cases where *breseq* has been applied to sets of evolutionarily related genomes and when specific mutation predictions have been experimentally validated that its accuracy and sensitivity have been found to be similar to what was obtained with the simulated data sets (>95%) for the types of SV that it predicts [5, 22].

**Figure 10:**
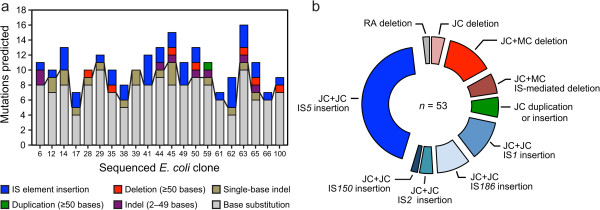
**Reanalysis of evolved** ***E. coli*** **samples for structural variation. a)** Summary of mutations predicted by *breseq* in 21 clones sequenced after 6,000 generations of growth in a mutation accumulation experiment [19]. These samples were previously analyzed for single-base substitutions and small indels. The line extending across the bars separates single-base substitutions and indels from mutations affecting more bases that were classified as structural variants. Full details for all mutations predicted in the ancestor of this experiment and each evolved lineage are provided in Additional file 2. **b)** Overall representation of the different types of structural variants predicted from combinations of new junction (JC) and missing coverage (MC) evidence across all 21 genomes. One structural variant was predicted from spurious read alignment (RA) evidence, as described in the text.

Overall, we found that 24% of the 225 mutations predicted in the mutation accumulation experiment led to structural variation in the *E. coli* chromosome (Figure 10, Additional file 2). Insertions of new copies of insertion sequence elements, particularly IS*5*, dominated among these events. This reanalysis enables us to estimate a spontaneous rate of SV mutations, which accounts for all mutations that are not single-base substitutions or short indels, in *E. coli* K-12 MG1655 of 0.00042 mutations per genome per generation (with a Poisson 95% confidence interval from 0.00032–0.00055). Of the SV predicted, 92% of the events were fully predicted by *breseq* without the need for any manual examination of “orphan” pieces of evidence to resolve them into precise molecular events. Thus, *breseq* is a useful tool for discovering mutations that cause structural variation in order to more comprehensively understand how microbial genomes evolve.

### Planned future directions

Metagenomic resequencing data, generated from a mixture of haploid microbial genomes that have evolved from a common ancestor, can currently be analyzed by *breseq* to estimate the frequencies of single-base substitution and indel variants in a population [21, 29, 38]. We intend to extend this capability, leveraging the work described here, to predict the frequencies of polymorphic structural variants in mixed population samples. The frequencies of mutations involving new junctions can be estimated by counting sequencing reads that span new junction candidates versus those that support unmutated reference genome sequences [5, 39]. This procedure could potentially detect structural variants at much lower frequencies than single-nucleotide or indel variants, as the occurrence of chimeric reads generating false-positive evidence for such mutations may be much rarer than the rate of base errors in sequencing data, but this hypothesis remains to be tested.

We also plan to have *breseq* integrate additional kinds of evidence from more fully analyzing short-read DNA sequencing data to make new and more precise mutation predictions. We have shown that split-read alignment information is very effective for predicting SV and other mutations in haploid microbial genomes, in agreement with other studies [11, 12]. However, using paired-end read mapping and analyzing “discordant pair” evidence could further aid in resolving *breseq* mutation predictions in or near repetitive genomic regions. New types of SV, such as large gene duplications, could also be predicted without user intervention in the future by examining anomalies in read-depth coverage across the reference genome to create “copy number” evidence. This analysis might use a circular binary segmentation algorithm [40] after accounting for biases in read-depth coverage based on DNA fragment GC-content [41], location in a chromosome with respect to an origin of DNA replication [42], and other factors.

Finally, we intend to further improve *breseq*’s ability to fully resolve and annotate complex events that may involve successive mutations. For example, we commonly observe genomes where a first IS insertion was followed by a second, adjacent IS-mediated deletion in genomes derived from the Lenski long-term laboratory evolution experiment with *E. coli* [5]. Currently, these compound events must be manually annotated as two mutations from a combination of evidence consisting of two new sequence junctions and one missing coverage interval.

## Conclusion

Structural variation in microbial genomes is a common source of genetic diversity that can be important during strain evolution inside and outside the laboratory. Currently, *ad hoc* or non-automated approaches with unproven performance are often used to detect SV in microbial genome resequencing data sets, or SV is neglected entirely and only single-nucleotide and short indel variation are analyzed. We have demonstrated that *breseq* can fully predict many types of SV in clonal samples from information about regions of a reference genome joined by split-read alignments and regions lacking sequencing coverage. For example, we found that 24% of the mutations detected in a *breseq* reanalysis of genomes from an *E. coli* K-12 evolution experiment resulted in SV, with new insertions of transposable elements dominating among these major chromosomal changes. The SV-prediction capability of *breseq* should be useful for more fully characterizing genetic diversity in similar studies of evolving microorganisms.

## Availability and requirements

**Project name:***breseq*

**Project home page:**http://barricklab.org/breseq

**Operating systems:** Linux, Mac OSX, Windows (Cygwin)

**Programming languages:** C++, R

**Other requirements:** Bowtie 2 version 2.1.0 or higher, R version 2.14 or higher

**License:** GNU General Public License (version 2)

**Any restrictions to use by non-academics:** none

## Electronic supplementary material

Additional file 1: **[breseq-0.25.tar.gz].** Source code archive for *breseq* computational pipeline (version 0.25). (ZIP 9 MB)

Additional file 2: **[MA_experiment_mutation_predictions.zip].** Archive detailing *breseq* predictions of mutations in genomes from the *E. coli* mutation accumulation (MA) experiment. File contents are as follows: **ancestor.gd**, differences between the ancestor strain of the MA experiment and the *E. coli* MG1655 reference genome (GenBank:NC_000913.2) in Genome Diff format. This text-based file format is described in the *breseq* documentation; **ancestor.gff**, an annotated genome sequence of the MA ancestor in GFF3 format, constructed by applying these mutations to the *E. coli* reference sequence; Other *.gd files, Genome Diff files describing mutations predicted in each of the 21 clones that were resequenced after 6,000 generations of evolution in the MA experiment. (ZIP 2 MB)

## Abbreviations

JC: New junction evidence
MC: Missing coverage evidence
SNV: Single-nucleotide variation
SV: Structural variation
SRA: Split-read alignment
PEM: Paired-end read mapping.
