# Supplementary material for: Identifying structural variation in haploid microbial genomes from short-read resequencing data using breseq
Source: BMC Genomics. 2014 Nov 29;15(1):1039. doi: 10.1186/1471-2164-15-1039 (PMC4300727; doi:10.1186/1471-2164-15-1039)
Supplement: Supplementary file 1 — Additional file 1: [breseq-0.25.tar.gz]. Source code archive for breseq computational pipeline (version 0.25). (ZIP 9 MB) [file 12864_2014_6751_MOESM1_ESM.zip › breseq-0.25.tar.gz!/breseq-0.25/documentation/acknowledgments.html]

Acknowledgments — breseq 0.25 documentation


### Navigation

- index
- next |
- previous |
- breseq 0.25 documentation »

# Acknowledgments¶

## Developers¶

- Jeffrey E. Barrick
- Jordan Borges
- Geoffrey Colburn
- Daniel E. Deatherage
- Tyler Fields
- David B. Knoester
- Matt Strand
- Aaron Reba
- Austin G. Meyer
- Alex Hu

**breseq** was initially developed at Michigan State University while Jeff was a postdoc with Richard Lenski and Charles Ofria, and while David was a graduate student in the lab of Philip McKinley.

It is now developed in the Barrick lab in the Department of Molecular Biosciences at the University of Texas at Austin. Lab Website

## Testers¶

We thank Zachary Blount, Jim Bull, Tim Cooper, Grace Ellis, Tara Gianoulis, Aleeza Gerstein, Dan Goodman, J.P. Jerome, Megan Larsen, Ranno Nahku, Hanspieter Niederstrasser, Elizabeth Perry, Gerda Saxer, William Trimble, Sébastien Wielgoss, and participants in Titus Brown’s 2010 summer course in next-gen sequencing at the Kellogg Biological Station for providing feedback on **breseq** usage and datasets for testing.

## Funding¶

The following grants have supported development of **breseq**:

1. National Institutes of Health (2009-2010). Pathway to Independence Award [K99GM087550 to J.E.B.]
2. National Science Foundation (2007-2008). Postdoctoral Fellowship in Biological Informatics [DBI-0630687 to J.E.B.]
3. National Science Foundation (2010). BEACON Center at Michigan State University [DBI-0939454 subcontract to J.E.B].
4. University of Texas at Austin CPRIT Research Traineeship to D.E.D.

### Table Of Contents

- Acknowledgments
  - Developers
  - Testers
  - Funding

#### Previous topic

References

#### Next topic

**gdtools** Utility Program

### This Page

- Show Source

### Quick search


Enter search terms or a module, class or function name.

### Navigation

- index
- next |
- previous |
- breseq 0.25 documentation »

© Copyright 2014 Jeffrey E. Barrick and contributors.
Created using Sphinx 1.1.
