# Supplementary material for: Identifying structural variation in haploid microbial genomes from short-read resequencing data using breseq
Source: BMC Genomics. 2014 Nov 29;15(1):1039. doi: 10.1186/1471-2164-15-1039 (PMC4300727; doi:10.1186/1471-2164-15-1039)
Supplement: Supplementary file 1 — Additional file 1: [breseq-0.25.tar.gz]. Source code archive for breseq computational pipeline (version 0.25). (ZIP 9 MB) [file 12864_2014_6751_MOESM1_ESM.zip › breseq-0.25.tar.gz!/breseq-0.25/documentation/gd_format.html]

GenomeDiff Format — breseq 0.25 documentation


### Navigation

- index
- previous |
- breseq 0.25 documentation »

# **GenomeDiff** Format¶

The **GenomeDiff** file format describes mutational differences between a reference DNA sequence and a sample. It may also include evidence from computational analysis or experiments that supports mutations.

An example of a portion of a **GenomeDiff** file:

```
#=GENOME_DIFF 1.0
DEL  61      11      NC_001416       139     1
INS  62      12      NC_001416       14266   G
SNP  63      13      NC_001416       20661   G
INS  64      14      NC_001416       20835   C
SNP  65      15      NC_001416       21714   A
DEL  60      33,1    NC_001416       21738   5996
SNP  66      35      NC_001416       31016   C
...
MC   9               NC_001416       1       2       0       0       left_inside_cov=0       left_outside_cov=NA     right_inside_cov=0      right_outside_cov=169
RA   11              NC_001416       139     0       G       .       frequency=1     new_cov=34/40   quality=309.0   ref_cov=0/0     tot_cov=34/40
JC   2               NC_001416       5491    1       NC_001416       30255   1       0       alignment_overlap=4     coverage_minus=8        coverage_plus=0 flanking_left=35        flanking_right=35       key=NC_001416__5491__1__NC_001416__30251__1__4____35__35__0__0  max_left=30     max_left_minus=30       max_left_plus=0 max_min_left=0  max_min_left_minus=0    max_min_left_plus=0     max_min_right=11        max_min_right_minus=11  max_min_right_plus=0    max_right=11    max_right_minus=11      max_right_plus=0        min_overlap_score=44    pos_hash_score=7        reject=NJ,COV   side_1_annotate_key=gene        side_1_overlap=4        side_1_redundant=0      side_2_annotate_key=gene        side_2_overlap=0        side_2_redundant=0      total_non_overlap_reads=8       total_reads=8
JC   3               NC_001416       13180   1       NC_001416       13218   1       0       alignment_overlap=4     coverage_minus=1        coverage_plus=0 flanking_left=35        flanking_right=35       key=NC_001416__13180__1__NC_001416__13214__1__4____35__35__0__0 max_left=17     max_left_minus=17       max_left_plus=0 max_min_left=0  max_min_left_minus=0    max_min_left_plus=0     max_min_right=14        max_min_right_minus=14  max_min_right_plus=0    max_right=14    max_right_minus=14      max_right_plus=0        min_overlap_score=14    pos_hash_score=1        reject=NJ,COV   side_1_annotate_key=gene        side_1_overlap=4        side_1_redundant=0      side_2_annotate_key=gene        side_2_overlap=0        side_2_redundant=0      total_non_overlap_reads=1       total_reads=1
RA   12              NC_001416       14266   1       .       G       frequency=1     new_cov=44/31   quality=186.3   ref_cov=0/0     tot_cov=44/31
JC   5               NC_001416       14869   -1      NC_001416       15609   -1      0       alignment_overlap=7     coverage_minus=1        coverage_plus=0 flanking_left=35        flanking_right=35       key=NC_001416__14869__0__NC_001416__15616__0__7____35__35__0__0 max_left=21     max_left_minus=21       max_left_plus=0 max_min_left=0  max_min_left_minus=0    max_min_left_plus=0     max_min_right=7 max_min_right_minus=7   max_min_right_plus=0    max_right=7     max_right_minus=7       max_right_plus=0        min_overlap_score=7     pos_hash_score=1        reject=NJ,COV   side_1_annotate_key=gene        side_1_overlap=7        side_1_redundant=0      side_2_annotate_key=gene        side_2_overlap=0        side_2_redundant=0      total_non_overlap_reads=1       total_reads=1
```

## Format specification¶

### Version line¶

The first line of the file must define that this is a **GenomeDiff** file and the version of the file specification used:

```
#=GENOME_DIFF 1.0
```

### Metadata lines¶

Lines beginning with **#=<name> <value>** are interpreted as metadata. (Thus, the first line is assigning a metadata item named GENOME\_DIFF a value of 1.0.) Names cannot include whitespace characters. Values may include whitespace characters. Lines with the same name are concatenated with single spaces added between them.

### Comment lines¶

Lines beginning with whitespace and # are comments. Comments may not occur at the end of a data line.

### Data lines¶

Data lines describe either a mutation or evidence from an analysis that can potentially support a mutational event. Data fields are tab-delimited. Each line begins with several fields containing information common to all types, continues with a fixed number of type-specific fields, and ends with an arbitrary number of name=value pairs that store optional information.

1. **type** *<string>*

   type of the entry on this line.
2. **id or evidence-id** *<uint32>*

   For evidence and validation lines, the id of this item. For mutation lines, the ids of all evidence or validation items that support this mutation. May be set to ‘.’ if a line was manually edited.
3. **parent-ids** *<uint32>*

   ids of evidence that support this mutation. May be set to ‘.’ or left blank.

*mutation* types are 3 letters: SNP, SUB, DEL, INS, MOB, AMP, CON, INV.

*evidence* types are 2 letters: RA, MC, JC, UN.

*validation* types are 4 letters: TSEQ, PFLP, RFLP, PFGE, PHYL, CURA.

### Mutational Event Types¶

#### SNP: Base substitution mutation¶

4. **seq\_id** *<string>*

   id of reference sequence fragment containing mutation, evidence, or validation.
5. **position** *<uint32>*

   position in reference sequence fragment of base to replace.
6. **new\_seq** *<char>*

   new base at position.

#### SUB: Multiple base substitution mutation¶

4. **seq\_id** *<string>*

   id of reference sequence fragment containing mutation, evidence, or validation.
5. **position** *<uint32>*

   position in the reference sequence of the first base that will be replaced.
6. **size** *<uint32>*

   number of bases *after* the specified reference position to replace with **new\_seq**.
7. **new\_seq** *<string>*

   new bases to substitute.

#### DEL: Deletion mutation¶

4. **seq\_id** *<string>*

   id of reference sequence fragment containing mutation, evidence, or validation.
5. **position** *<uint32>*

   position in reference sequence fragment of first deleted base.
6. **size** *<uint32>*

   number of bases deleted in reference.

##### Additional DEL named fields¶

- **mediated=***<mobile\_element\_family>*
  :   This deletion appears to be mediated by a molecular event involving a mobile element such as a transposon. A copy of the mobile element is found on the boundary of the deleted region and a new junction at the opposite end of the deletion matches the end of the mobile element.
- **between=***<repeat\_family>*
  :   This deletion appears to result from homologous recombination or polymerase slipping between two existing copies of the same genomic repeat (e.g. tRNA, IS element) in the genome. One copy of the repeat is deleted by this event.
- **repeat\_seq=***<string>*, **repeat\_length=***<uint32>*, **repeat\_ref\_num=***<uint32>*, **repeat\_new\_copies=***<uint32>*
  :   This deletion is in a short sequence repeat consisting of tandem copies of **repeat\_seq** repeated **repeat\_ref\_num** times in the ancestor and **repeat\_new\_copies** after a mutation. To be annotated in this way the copy of the repeat in the reference genome must consist of at least two repeat copies and have a length of five of more total bases (**repeat\_length** × **repeat\_ref\_num** ≥ 5).

#### INS: Insertion mutation¶

4. **seq\_id** *<string>*

   id of reference sequence fragment containing mutation, evidence, or validation.
5. **position** *<uint32>*

   position in reference sequence fragment. New bases are inserted *after* this position.
6. **new\_seq** *<string>*

   new bases to be inserted in the reference.

##### Additional INS named fields¶

- **repeat\_seq=***<string>*, **repeat\_length=***<uint32>*, **repeat\_ref\_num=***<uint32>*, **repeat\_new\_copies=***<uint32>*
  :   This insertion is in a short sequence repeat consisting of tandem copies of **repeat\_seq** repeated **repeat\_ref\_num** times in the ancestor and **repeat\_new\_copies** after a mutation. To be annotated in this way the copy of the repeat in the reference genome must consist of at least two repeat copies and have a length of five of more total bases (**repeat\_length** × **repeat\_ref\_num** ≥ 5).
- **insert\_position=***<uint32>*
  :   Used when there are multiple insertion events after the same reference base to order the insertions. This typically happens in polymorphism mode and when manually breaking up an insertion of bases into distinct mutational events. Numbering of insertions begins with 1.

#### MOB: Mobile element insertion mutation¶

4. **seq\_id** *<string>*

   id of reference sequence fragment containing mutation, evidence, or validation.
5. **position** *<uint32>*

   position in reference sequence fragment of the first duplicated base at the target site.
6. **repeat\_name** *<string>*

   name of the mobile element. Should correspond to an annotated **repeat\_region** or **mobile\_element** feature in the reference sequence.
7. **strand** *<1/-1>*

   strand of mobile element insertion.
8. **duplication\_size** *<uint32>*

   number of target site bases duplicated during insertion of the mobile element, beginning with the specified reference position. If the value of this field is negative, then it indicates that the absolute value of this number of bases were deleted at the target site beginning with the specified position. If the value of this field is zero, then the there were no duplicated bases, and the mobile element was inserted after the specified base position.

##### Additional MOB named fields¶

- **del\_start=***<uint32>*, **del\_end=***<uint32>*

  > Delete this many bases from the start or end of the inserted mobile element. This deletion occurs with respect to the top strand of the genome after the element is flipped to the orientation with which it will be inserted.
- **ins\_start=***<string>*, **ins\_end=***<string>*

  > Append the specified bases to the start or end of the inserted mobile element. These insertions occur after any deletions and will be inside of any duplicated target site bases.
- **mob\_region**=*<seq\_id:start-end >*

  > Use the existing copy of the mobile element specified as a seq\_id:start-end region to apply this mutation. Useful when different annotated members of a mobile element family have slightly different sequences.

#### AMP: Amplification mutation¶

4. **seq\_id** *<string>*

   id of reference sequence fragment containing mutation, evidence, or validation.
5. **position** *<uint32>*

   position in reference sequence fragment.
6. **size** *<uint32>*

   number of bases duplicated starting with the specified reference position.
7. **new\_copy\_number** *<uint32>*

   new number of copies of specified bases.

##### Additional AMP named fields¶

- **mediated=***<mobile\_element\_family>*, **mediated\_strand=***<1/-1>*
  :   This amplification appears to be mediated by a mobile element insertion. New copies of the mobile element are inserted in the specified orientation before each new copy of the amplified region.

#### CON: Gene conversion mutation¶

4. **seq\_id** *<string>*

   id of reference sequence fragment containing mutation, evidence, or validation.
5. **position** *<uint32>*

   position in reference sequence fragment that was the target of gene conversion from another genomic location.
6. **size** *<uint32>*

   number of bases to replace in the reference genome beginning at the specified position.
7. **region** *<sequence:start-end>*

   Region in the reference genome to use as a replacement.

#### INV: Inversion mutation¶

4. **seq\_id** *<string>*

   id of reference sequence fragment containing mutation, evidence, or validation.
5. **position** *<uint32>*

   position in reference sequence fragment.
6. **size** *<uint32>*

   number of bases in inverted region beginning at the specified reference position.

### Standard name=value pairs¶

#### Counting Mutations¶

These attributes control how molecular events in a **GenomeDiff** are counted for summary purposes.

- **between**=*<element\_name>*

  > This mutation occurs between copies of this element. For example, a deletion caused by recombination between two copies of a mobile element.
- **mediated**=*<element\_name>*

  > This mutation was mediated by insertion of a new copy of this element and recombination with an existing copy, such that the number of this element did not net increase in the resulting genome.
- **adjacent**=*<element\_name>*

  > This mutation is adjacent to the specified element. For example, it may be an insertion of a base next to a mobile element. We may want to ignore mutations in this category because they represent a hotspot with an atypical mutation rate.
- **with**=*<mutation\_id>*

  > This mutation should not be counted separately. It should be counted as a **single** molecular event with the other specified mutation (which does not need a with tag)

#### Applying Mutations¶

These advanced attributes control how mutations are applied when using **gdtools APPLY** to build a new reference genome from the original reference genome and a **GenomeDiff** and when building phylogenetic trees from multiple samples. They are not generated automatically by **breseq**.

- **before**=*<mutation\_id>*

  > Apply this mutation before another mutation. For example, did a base substitution occur after a region was duplicated, thus it is only in one copy or did it occur before the duplication, thus altering both copies? Did a base substitution happen before a deletion, hiding a mutation that should be included in any phylogenetic inference? When this attributes is present, mutations will be applied in order according to their genomic positions.
- **within**=*<mutation\_id>* or **within**=*<mutation\_id>:<copy\_index>*

  > This mutation happens within a different mutation. These options can specify, for example, that a base substitution happens in the second copy of a duplicated region. If *<mutation\_id>* refers to an AMP, then it must be of the form *<mutation\_id>:<copy\_index>* and the mutation is placed in the corresponding copy of the specified coordinates. If *<mutation\_id>* refers to an INS, then it must be of the form *<mutation\_id>* with no *<copy\_index>*, and the position of the mutation will be interpreted as happening after the INS bases are inserted, so that it can change those new bases. If *<mutation\_id>* refers to a MOB with no *<copy\_index>*, then the mutation is placed within the newly inserted sequence of the mobile element with the position of the mutation interpreted as happening after those bases are inserted. If it refers to a MOB and is of the form *<mutation\_id>:<copy\_index>*, then the mutation is placed within the specified copy of the target site duplication.
- **deleted**=*1*

  > The sequence change caused by this mutation was made irrelevant by subsequent mutations that deleted or further changed the affected region. Annotation of this mutation in the given genome was inferred based on phylogeny. It will not be applied when generating the mutated genome.

### Evidence Types¶

#### RA: Read alignment evidence¶

Line specification:

4. **seq\_id** *<string>*

   id of reference sequence fragment containing mutation, evidence, or validation.
5. **position** *<uint32>*

   position in reference sequence fragment.
6. **insert\_position** *<uint32>*

   number of bases inserted after the reference position to get to this base. An value of zero refers to the base. A value of 5 means that this evidence if for the fifth newly inserted column after the reference position.
7. **ref\_base** *<char>*

   base in the reference genome.
8. **new\_base** *<char>*

   new base supported by read alignment evidence.

#### MC: Missing coverage evidence¶

Line specification:

4. **seq\_id** *<string>*

   id of reference sequence fragment containing mutation, evidence, or validation.
5. **start** *<uint32>*

   start position in reference sequence fragment.
6. **end** *<uint32>*

   end position in reference sequence of region.
7. **start\_range** *<uint32>*

   number of bases to offset *after* the **start position** to define the upper limit of the range where the start of a deletion could be.
8. **end\_range** *<uint32>*

   number of bases to offset *before* the **end position** to define the lower limit of the range where the start of a deletion could be.

Essentially this is evidence of missing coverage between two positions in the ranges [start, start+start\_range] [end-end\_range, end].

#### JC: New junction evidence¶

4. **side\_1\_seq\_id** *<string>*

   id of reference sequence fragment containing side 1 of the junction.
5. **side\_1\_position** *<uint32>*

   position of side 1 at the junction boundary.
6. **side\_1\_strand** *<1/-1>*

   direction that side 1 continues matching the reference sequence
7. **side\_2\_seq\_id** *<string>*

   id of reference sequence fragment containing side 2 of the junction.
8. **side\_2\_position** *<uint32>*

   position of side 2 at the junction boundary.
9. **side\_2\_strand** *<1/-1>*

   direction that side 2 continues matching the reference sequence.

9. **overlap** *<uint32>*

   Number of bases that the two sides of the new junction have in common.

#### UN: Unknown base evidence¶

Line specification:

4. **seq\_id** *<string>*

   id of reference sequence fragment containing mutation, evidence, or validation.
5. **start** *<uint32>*

   start position in reference sequence of region.
6. **end** *<uint32>*

   end position in reference sequence of region.

### Validation Types¶

These items indicate that mutations have been validated by further, targeted experiments.

#### CURA: True-positive curated by an expert¶

An expert has examined the data output from a prediction program and determined that this mutations is a true positive.

Line specification:

4. **expert** *<string>*

   Name or initials of the person who predicted the mutation.

#### FPOS: False-positive curated by an expert¶

An expert has examined the raw read data and determined that this predicted mutation is a false positive.

Line specification:

4. **expert** *<string>*

   Name or initials of the person who predicted the mutation.

#### PHYL: Phylogenetic comparison¶

This validation was transferred from validation in another, related genome.

Line specification:

4. **gd** *<string>*

   Name of the genome\_diff file containing the evidence.

#### TSEQ: Targeted re-sequencing¶

Line specification:

4. **seq\_id** *<string>*

   id of reference sequence fragment containing mutation, evidence, or validation.
5. **primer1\_start** *<uint32>*

   position in reference sequence of the 5’ end of primer 1.
6. **primer1\_end** *<uint32>*

   position in reference sequence of the 3’ end of primer 1.
7. **primer2\_start** *<uint32>*

   position in reference sequence of the 5’ end of primer 2.
8. **primer2\_end** *<uint32>*

   position in reference sequence of the 3’ end of primer 2.

For primer 1, start < end. For primer 2, end < start.

#### PFLP: PCR-fragment length polymorphism¶

Line specification:

4. **seq\_id** *<string>*

   id of reference sequence fragment containing mutation, evidence, or validation.
5. **primer1\_start** *<uint32>*

   position in reference sequence of the 5’ end of primer 1.
6. **primer1\_end** *<uint32>*

   position in reference sequence of the 3’ end of primer 1.
7. **primer2\_start** *<uint32>*

   position in reference sequence of the 5’ end of primer 2.
8. **primer2\_end** *<uint32>*

   position in reference sequence of the 3’ end of primer 2.

For primer 1, start < end. For primer 2, end < start.

#### RFLP: Restriction fragment length polymorphism¶

Line specification:

4. **seq\_id** *<string>*

   id of reference sequence fragment containing mutation, evidence, or validation.
5. **primer1\_start** *<uint32>*

   position in reference sequence of the 5’ end of primer 1.
6. **primer1\_end** *<uint32>*

   position in reference sequence of the 3’ end of primer 1.
7. **primer2\_start** *<uint32>*

   position in reference sequence of the 5’ end of primer 2.
8. **primer2\_end** *<uint32>*

   position in reference sequence of the 3’ end of primer 2.
9. **enzyme** *<string>*

   Restriction enzyme used to distinguish reference from mutated allele.

For primer 1, start < end. For primer 2, end < start.

#### PFGE: Pulsed-field gel electrophoresis¶

Changes in fragment sizes of genomic DNA digested with restriction enzymes and separated by pulsed-field

Line specification:

4. **seq\_id** *<string>*

   id of reference sequence fragment containing mutation, evidence, or validation.
5. **restriction enzyme** *<string>*

> Restriction enzyme used to digest genomic DNA and observe fragments.

#### NOTE: Note¶

Generic container for a note about a mutation prediction

Line specification:

4. **note** *<string>*

   Free text note.

#### MASK: Repeat mask a section¶

Artificially mask a section of DNA as “N”s. This is useful for creating modified reference sequences, particularly for targeted sequencing approaches.
Line specification:

4. **seq\_id** *<string>*

   id of reference sequence fragment containing mutation, evidence, or validation.
5. **position** *<uint32>*

   position in reference sequence fragment.
6. **size** *<uint32>*

   number of bases masked to “N” in reference, including reference position.

### Table Of Contents

- **GenomeDiff** Format
  - Format specification
    - Version line
    - Metadata lines
    - Comment lines
    - Data lines
    - Mutational Event Types
      - SNP: Base substitution mutation
      - SUB: Multiple base substitution mutation
      - DEL: Deletion mutation
        - Additional DEL named fields
      - INS: Insertion mutation
        - Additional INS named fields
      - MOB: Mobile element insertion mutation
        - Additional MOB named fields
      - AMP: Amplification mutation
        - Additional AMP named fields
      - CON: Gene conversion mutation
      - INV: Inversion mutation
    - Standard name=value pairs
      - Counting Mutations
      - Applying Mutations
    - Evidence Types
      - RA: Read alignment evidence
      - MC: Missing coverage evidence
      - JC: New junction evidence
      - UN: Unknown base evidence
    - Validation Types
      - CURA: True-positive curated by an expert
      - FPOS: False-positive curated by an expert
      - PHYL: Phylogenetic comparison
      - TSEQ: Targeted re-sequencing
      - PFLP: PCR-fragment length polymorphism
      - RFLP: Restriction fragment length polymorphism
      - PFGE: Pulsed-field gel electrophoresis
      - NOTE: Note
      - MASK: Repeat mask a section

#### Previous topic

**gdtools** Utility Program

### This Page

- Show Source

### Quick search


Enter search terms or a module, class or function name.

### Navigation

- index
- previous |
- breseq 0.25 documentation »

© Copyright 2014 Jeffrey E. Barrick and contributors.
Created using Sphinx 1.1.
