# Supplementary material for: Identifying structural variation in haploid microbial genomes from short-read resequencing data using breseq
Source: BMC Genomics. 2014 Nov 29;15(1):1039. doi: 10.1186/1471-2164-15-1039 (PMC4300727; doi:10.1186/1471-2164-15-1039)
Supplement: Supplementary file 1 — Additional file 1: [breseq-0.25.tar.gz]. Source code archive for breseq computational pipeline (version 0.25). (ZIP 9 MB) [file 12864_2014_6751_MOESM1_ESM.zip › breseq-0.25.tar.gz!/breseq-0.25/documentation/gd_usage.html]

gdtools Utility Program — breseq 0.25 documentation


### Navigation

- index
- next |
- previous |
- breseq 0.25 documentation »

# **gdtools** Utility Program¶

Performs various functions on **GenomeDiff** formatted files. Options depend on the COMMAND supplied. Only a small subset of these commands are described below.
For a full list of **gdtools** subcommands run it from the command line with no options:

```
gdtools
```

## Command: ANNOTATE (or COMPARE)¶

Usage:

```
gdtools ANNOTATE [-o annotated.html] -r reference.gbk input.1.gd [input.2.gd ... ]
```

Annotate a **GenomeDiff** file with information about mutations (what genes they affect, amino acid substitutions, etc.)
Default output is to another **GenomeDiff**, but an HTML table can be produced with a table of mutations in a single **GenomeDiff**
file or to compare the mutations present in several **GenomeDiff** files. This subcommand can be called as ANNOTATE or COMPARE. Both
have the same effect.

-r <file\_path>, --reference=<file\_path>¶
:   Reference sequence files (Genbank, GFF, or FASTA). This option may be entered multiple times. REQUIRED

-o <file\_path>, --output=<file\_path>¶
:   File name for the output|Genome Diff| or HTML. DEFAULT: “annotated.gd” or “annotated.html”.

--html¶
:   Output an HTML table instead of a **GenomeDiff** file.

input1.gd input2.gd ...
:   Input **GenomeDiff** file(s). This option may be entered multiple times to compare across files. REQUIRED

Warning

Some advanced **GenomeDiff** attributes for mutations, such as **within** and **before**, are ignored when generating compare tables.

## Command: APPLY¶

Usage:

```
gdtools APPLY [ -o output.gff3 -f GFF3 ] -r reference.gbk input.gd
```

Apply the mutations described in the input **GenomeDiff** to the reference sequence(s).

-r <file\_path>, --reference=<file\_path>¶
:   Reference sequence files (Genbank, GFF, or FASTA). This option may be entered multiple times. REQUIRED

-o <file\_path>, --output=<file\_path>¶
:   Output file containing the muta. DEFAULT: “output.\*”

-f <output\_format>, --format=<output\_format>¶
:   Output format. Possible values are “fasta” or “gff3”.

input.gd
:   Input **GenomeDiff** file. REQUIRED

### Table Of Contents

- **gdtools** Utility Program
  - Command: ANNOTATE (or COMPARE)
  - Command: APPLY

#### Previous topic

Acknowledgments

#### Next topic

**GenomeDiff** Format

### This Page

- Show Source

### Quick search


Enter search terms or a module, class or function name.

### Navigation

- index
- next |
- previous |
- breseq 0.25 documentation »

© Copyright 2014 Jeffrey E. Barrick and contributors.
Created using Sphinx 1.1.
