# Supplementary material for: Identifying structural variation in haploid microbial genomes from short-read resequencing data using breseq
Source: BMC Genomics. 2014 Nov 29;15(1):1039. doi: 10.1186/1471-2164-15-1039 (PMC4300727; doi:10.1186/1471-2164-15-1039)
Supplement: Supplementary file 1 — Additional file 1: [breseq-0.25.tar.gz]. Source code archive for breseq computational pipeline (version 0.25). (ZIP 9 MB) [file 12864_2014_6751_MOESM1_ESM.zip › breseq-0.25.tar.gz!/breseq-0.25/documentation/genindex.html]

Index — breseq 0.25 documentation


### Navigation

- index
- breseq 0.25 documentation »

# Index

**Symbols**
| **B**
| **G**

## Symbols

|  |  |
| --- | --- |
| --format <PNG/PDF>  breseq command line option  --html  gdtools-ANNOTATE-(or-COMPARE) command line option  --max-rejected-polymorphisms-to-show <int>  breseq command line option  --no-junction-prediction  breseq command line option  --polymorphism-bias-p-value-cutoff <double>  breseq command line option  --polymorphism-coverage-both-strands <int>  breseq command line option  --polymorphism-frequency-cutoff <double>  breseq command line option  --polymorphism-log10-e-value-cutoff <float>  breseq command line option  --polymorphism-prediction  breseq command line option  --polymorphism-reject-homopolymer-length <int>  breseq command line option  --resolution <int>  breseq command line option  -1, --total-only  breseq command line option  -b <file\_path>, --bam <file\_path>  breseq command line option, [1] | -f <file\_path>, --fasta <file\_path>  breseq command line option, [1]  -f <output\_format>, --format=<output\_format>  gdtools-APPLY command line option  -j <int>, --num-processors <int>  breseq command line option  -n <int>, --max-reads <int>  breseq command line option  -n <string>, --name <string>  breseq command line option  -o <file\_path>, --output=<file\_path>  gdtools-ANNOTATE-(or-COMPARE) command line option  gdtools-APPLY command line option  -o <path>, --output <path>  breseq command line option, [1]  -p, --predict-polymorphisms  breseq command line option  -r <file\_path>, --reference <file\_path>  breseq command line option  -r <file\_path>, --reference=<file\_path>  gdtools-ANNOTATE-(or-COMPARE) command line option  gdtools-APPLY command line option  -r <region> , --region <region> , --unnamed-arguments-- <region> [<region2>, <region2>, ...]  breseq command line option  -r <region>, --region <region>, --unnamed-arguments-- <region> [<region2>, <region2>, ...]  breseq command line option  -t, --table  breseq command line option |

## B

breseq command line option
:   --format <PNG/PDF>

    --max-rejected-polymorphisms-to-show <int>

    --no-junction-prediction

    --polymorphism-bias-p-value-cutoff <double>

    --polymorphism-coverage-both-strands <int>

    --polymorphism-frequency-cutoff <double>

    --polymorphism-log10-e-value-cutoff <float>

    --polymorphism-prediction

    --polymorphism-reject-homopolymer-length <int>

    --resolution <int>

    -1, --total-only

    -b <file\_path>, --bam <file\_path>, [1]

    -f <file\_path>, --fasta <file\_path>, [1]

    -j <int>, --num-processors <int>

    -n <int>, --max-reads <int>

    -n <string>, --name <string>

    -o <path>, --output <path>, [1]

    -p, --predict-polymorphisms

    -r <file\_path>, --reference <file\_path>

    -r <region> , --region <region> , --unnamed-arguments-- <region> [<region2>, <region2>, ...]

    -r <region>, --region <region>, --unnamed-arguments-- <region> [<region2>, <region2>, ...]

    -t, --table

## G

|  |  |
| --- | --- |
| gdtools-ANNOTATE-(or-COMPARE) command line option  --html  -o <file\_path>, --output=<file\_path>  -r <file\_path>, --reference=<file\_path> | gdtools-APPLY command line option  -f <output\_format>, --format=<output\_format>  -o <file\_path>, --output=<file\_path>  -r <file\_path>, --reference=<file\_path> |

### Quick search


Enter search terms or a module, class or function name.

### Navigation

- index
- breseq 0.25 documentation »

© Copyright 2014 Jeffrey E. Barrick and contributors.
Created using Sphinx 1.1.
