# Supplementary material for: Identifying structural variation in haploid microbial genomes from short-read resequencing data using breseq
Source: BMC Genomics. 2014 Nov 29;15(1):1039. doi: 10.1186/1471-2164-15-1039 (PMC4300727; doi:10.1186/1471-2164-15-1039)
Supplement: Supplementary file 1 — Additional file 1: [breseq-0.25.tar.gz]. Source code archive for breseq computational pipeline (version 0.25). (ZIP 9 MB) [file 12864_2014_6751_MOESM1_ESM.zip › breseq-0.25.tar.gz!/breseq-0.25/documentation/index.html]

breseq Manual — breseq 0.25 documentation


### Navigation

- index
- next |
- breseq 0.25 documentation »

# **breseq** Manual¶

Contents:

- Introduction
- Installation
  - 1. Download source code
  - 2. Install external dependencies
    - MacOSX Instructions
  - 3. Compile and install **breseq**
    - Installing in a system-wide location
    - Installing in the source directory
    - Installing in a custom location
  - Common installation problems
    - Dependencies installed in custom locations
    - Other problems
  - Developers
- Usage Summary
  - **breseq**
  - Command: bam2aln
  - Command: bam2cov
- Test Drive
  - 1. Download data files
    - Reference sequence
    - Read files
  - 2. Run **breseq**
  - 3. Open **breseq** output
- Methods
  - Read Mapping
  - New junction evidence (JC)
    - Identifying candidate junctions
    - Scoring and accepting junctions
  - Read alignment evidence (RA)
    - Read end trimming
    - Base quality re-calibration
    - Haploid Bayesian SNP caller
    - Polymorphism Prediction
  - Unknown base evidence (UN)
  - Missing coverage evidence (MC)
    - Read coverage distribution
    - Seed and extend algorithm
  - Mutation prediction
    - Base substitutions
    - Short insertions and deletions
    - Large deletions
    - Mobile element insertions
    - Duplications
    - Other evidence
  - Limitations
- Output
  - HTML Archive
    - Mutation Display
    - Evidence Display
  - Processed Data
  - Viewing Output / Aligned Reads in the IGV
- References
- Acknowledgments
  - Developers
  - Testers
  - Funding

Appendices:

- gdtools Utility
- Genome Diff Format

Documentation last updated September 03, 2014.

#### Next topic

Introduction

### This Page

- Show Source

### Quick search


Enter search terms or a module, class or function name.

### Navigation

- index
- next |
- breseq 0.25 documentation »

© Copyright 2014 Jeffrey E. Barrick and contributors.
Created using Sphinx 1.1.
