# Supplementary material for: Identifying structural variation in haploid microbial genomes from short-read resequencing data using breseq
Source: BMC Genomics. 2014 Nov 29;15(1):1039. doi: 10.1186/1471-2164-15-1039 (PMC4300727; doi:10.1186/1471-2164-15-1039)
Supplement: Supplementary file 1 — Additional file 1: [breseq-0.25.tar.gz]. Source code archive for breseq computational pipeline (version 0.25). (ZIP 9 MB) [file 12864_2014_6751_MOESM1_ESM.zip › breseq-0.25.tar.gz!/breseq-0.25/documentation/installation.html]

Installation — breseq 0.25 documentation


### Navigation

- index
- next |
- previous |
- breseq 0.25 documentation »

# Installation¶

**breseq** is a command line tool implemented in C++ and R. It will compile and function on a variety of UNIX platforms, including MacOSX. **breseq** installation from the source code requires some basic familiarity with UNIX commands and environments.

Note

If you are unfamiliar with installing programs by compiling them from source code and you are working on MacOSX, then you should consider using the Fink package manager to automate installing breseq and the programs that it requires. More information about the Fink package for **breseq** is available here.

## 1. Download source code¶

The most recent **breseq** source code packages are available for download from Google Code.

## 2. Install external dependencies¶

Several external packages and software programs need to be installed to compile and use **breseq**:

- GCC (or other C++ compiler) already installed on many systems
- Bowtie2 (version 2.1.0 or higher) read mapping program
- R (version 2.1.4 or higher) statistical programming language

To install each dependency, use your system’s package manager or visit the respective web pages linked above and follow the instructions for your platform. More specific directions are available below for some platforms. You must make sure that the executables for **Bowtie2** and **R** are in your environment’s $PATH for **breseq** to function.

### MacOSX Instructions¶

You will need administrator privileges to install **breseq** dependencies using these instructions. We recommend that you install and use the package manager MacPorts to simplify these installation steps. Using **MacPorts** will generally take longer than downloading and installing the packages in other ways, but it greatly simplifies the searching you might otherwise have to do to track down all the prerequisites.

- **GCC**: Download and install Apple Developer tools (available from the App store).
- **Bowtie2**: Download and install the executable for your systems’s architecture from the Bowtie2 SourceForge site.

You can find out your system’s architecture using this command:

```
arch
```

It will return something like i386 or x86\_64.

You will need to move the executables to where your system can use them. If you change into the downloaded **Bowtie2** directory, you can use this command, for example:

```
sudo cp bowtie2* /usr/local/bin
```

If you type this command in the terminal:

```
which bowtie2
```

You should now get a message like this, telling you that the system can find your **Bowtie2** executable:

```
/usr/local/bin/bowtie2
```

- **R**: Download the installer package from http://www.r-project.org/.

## 3. Compile and install **breseq**¶

If you have admin privileges and want to install **breseq** in a standard location accessible to all users of a computer, then see *Installing in a system-wide location*. If you do not have admin privileges on your computer, then see *Installing in the source directory* or *Installing in a custom location*.

Note

If you encounter problems with one of the other install methods, we recommend that you try *Installing in the source directory*.

### Installing in a system-wide location¶

This method requires that you have admin privileges on your machine. After installation, all users of the machine will be able to run **breseq**.

Open a terminal window and change directory to the root of the **breseq** source distribution. Then, run these commands:

```
./configure
make
make test
sudo make install
```

make test is optional, but recommended. It should take less than 5 minutes to run and report success at the end if everything is operating correctly.

These commands compile and install not only **breseq**, but also some open-source code developed by others. These packages are included in the **breseq** source distribution under /extern:

- SAMtools

Warning

Installing **breseq** will overwrite any other versions of **SAMtools** that you have in the default ./configure install locations. To avoid this, you can follow the instructions in *Installing in the source directory* or *Installing in a custom location* to safely install **breseq** elsewhere.

### Installing in the source directory¶

This is the most robust way to install **breseq** if you do not have admin privileges on a system. All of the compiled programs and libraries will be self-contained in the original source tree.

Open a terminal window and change directory to the root of the **breseq** source distribution. Then, run these commands:

```
./configure --prefix=${PWD}
make
make test
make install
```

After installation, if you want to be able to call **breseq** commands without specifying the entire path to them, you will need to add the newly created “bin” directory within the **breseq** source to your $PATH.

For a **bash** shell you can usually use a command like this:

```
echo "export PATH=\$PATH:${PWD}/bin" >> ~/.bashrc
```

But the exact way to do this may depend on your system. Once you open a new terminal window so that it registers this change to your $PATH, you should be able to invoke **breseq** commands.

### Installing in a custom location¶

We’ll assume that you’ve chosen to install **breseq** in /mnt/home/me/local. Open a terminal window and change directory to the root of the **breseq** source distribution. Then, run these commands:

```
./configure --prefix=/mnt/home/me/local
make
make test
make install
```

This will create a usual UNIX grouping of program directories (with sub-directories like bin, lib, man, etc).

After installation, if you want to be able to call **breseq** commands without specifying the entire path to them, you will need to add the newly created “bin” directory within the **breseq** source to your $PATH.

For a **bash** shell you can usually use a command like this:

```
echo "export PATH=\$PATH:/mnt/home/me/local/bin" >> ~/.bashrc
```

But the exact way to do this may depend on your system. You may also want to similarly update your $MANPATH, $CPPFLAGS, $LD\_FLAGS, etc. Now you should be able to invoke **breseq** commands once you open a new terminal window.

## Common installation problems¶

### Dependencies installed in custom locations¶

In general, you will need to be sure that your environment is set up correctly to find and use each dependency. This will likely be taken care of for you if you use an installer package. If you install some dependencies from source or in custom locations, and run into problems with **breseq** installation, be sure to check that:

1. **R** is in your $PATH.
2. **Bowtie2** is in your $PATH.

Note

You may need to use absolute paths (i.e. /absolute/path) rather than paths relative to your home directory (i.e ~/path/relative/to/home) for these settings.

### Other problems¶

If you have a problem installing **breseq**, please send a detailed report to jeffrey.e.barrick@gmail.com.

## Developers¶

If you are working with a development version of **breseq** downloaded directly from the Google Code Mercurial repository, then you will need to run some additional commands and have additional tools installed in order to get it to compile or work with the XCode project.

These are detailed in the DEVELOPER text file found in the main directory of the source code.

### Table Of Contents

- Installation
  - 1. Download source code
  - 2. Install external dependencies
    - MacOSX Instructions
  - 3. Compile and install **breseq**
    - Installing in a system-wide location
    - Installing in the source directory
    - Installing in a custom location
  - Common installation problems
    - Dependencies installed in custom locations
    - Other problems
  - Developers

#### Previous topic

Introduction

#### Next topic

Usage Summary

### This Page

- Show Source

### Quick search


Enter search terms or a module, class or function name.

### Navigation

- index
- next |
- previous |
- breseq 0.25 documentation »

© Copyright 2014 Jeffrey E. Barrick and contributors.
Created using Sphinx 1.1.
