# Supplementary material for: Identifying structural variation in haploid microbial genomes from short-read resequencing data using breseq
Source: BMC Genomics. 2014 Nov 29;15(1):1039. doi: 10.1186/1471-2164-15-1039 (PMC4300727; doi:10.1186/1471-2164-15-1039)
Supplement: Supplementary file 1 — Additional file 1: [breseq-0.25.tar.gz]. Source code archive for breseq computational pipeline (version 0.25). (ZIP 9 MB) [file 12864_2014_6751_MOESM1_ESM.zip › breseq-0.25.tar.gz!/breseq-0.25/documentation/introduction.html]

Introduction — breseq 0.25 documentation


### Navigation

- index
- next |
- previous |
- breseq 0.25 documentation »

# Introduction¶

**breseq** (pronounced: *breeze-seq*) is a computational pipeline for the analysis of short-read re-sequencing data (e.g. 454, Illumina, SOLiD, etc.). It uses reference-based alignment approaches to predict mutations in a sample relative to an already sequenced genome. **breseq** is intended for microbial genomes (<10 Mb) and re-sequenced samples that are only slightly diverged from the reference sequence (<1 mutation per 1000 bp).

**breseq**‘s primary advantages over other existing software programs are that it can:

1. Predict new sequence junctions, such as those associated with mobile element insertions, from single-end read data.
2. Reliably identify short indel mutations by appropriately masking the ends of read alignments.
3. Produce annotated output describing biologically relevant mutational events.

**breseq** has been used to analyze data from the Lenski long-term evolution experiment with *E. coli* [Barrick2009a][Barrick2009b].

**breseq** should be generally useful to researchers who are:

1. Following mutations over time in microbial evolution experiments.
2. Checking strains for second-site mutations after genetic manipulations.
3. Identifying mutations that occur during strain improvement or after long-term culture of engineered strains.
4. Discovering what mutations arise in pathogens during infection or cause antibiotic resistance.

#### Previous topic

**breseq** Manual

#### Next topic

Installation

### This Page

- Show Source

### Quick search


Enter search terms or a module, class or function name.

### Navigation

- index
- next |
- previous |
- breseq 0.25 documentation »

© Copyright 2014 Jeffrey E. Barrick and contributors.
Created using Sphinx 1.1.
