# Supplementary material for: Identifying structural variation in haploid microbial genomes from short-read resequencing data using breseq
Source: BMC Genomics. 2014 Nov 29;15(1):1039. doi: 10.1186/1471-2164-15-1039 (PMC4300727; doi:10.1186/1471-2164-15-1039)
Supplement: Supplementary file 1 — Additional file 1: [breseq-0.25.tar.gz]. Source code archive for breseq computational pipeline (version 0.25). (ZIP 9 MB) [file 12864_2014_6751_MOESM1_ESM.zip › breseq-0.25.tar.gz!/breseq-0.25/documentation/methods.html]

Methods — breseq 0.25 documentation


### Navigation

- index
- next |
- previous |
- breseq 0.25 documentation »

# Methods¶

This section describes the algorithms used by **breseq**.

## Read Mapping¶

**breseq** uses Bowtie2 to map reads to the reference genome sequence:

Currently, **breseq** does not use the distance contraints available in paired-end or mate-paired libraries during read alignment or as a source of evidence supporting mutations. These data sets are treated as single-end reads.

**breseq** keeps track of two kinds of read alignments:

unique read matches
:   Where a read aligns best to only one location in the reference sequence.

repeat read matches
:   Where a read aligns equivalently to multiple locations in the reference sequence. Generally, these are long repeat regions.

For some calculations, **breseq** is concerned with:

unique-only reference positions
:   Position in the reference sequence that do not overlap any *repeat read matches*.

## New junction evidence (JC)¶

First, **breseq** searches for mosaic read alignments that may indicate new junctions in the sample between disjoint regions of the reference sequence.

### Identifying candidate junctions¶

In a pre-processing step, all read alignments with insertions or deletions of more than 2 bases are split into their constituent sub-alignments. This strategy tends to be more accurate than looking for these types of mutations as *Read alignment evidence (RA)* because gaps larger than a couple of bp can be problematic for generating accurate and consistent read alignments, especially for indels involving simple sequence repeats.

Next, for each read that has multiple alignments to the reference, all pairs of alignments are tested to find cases where:

1. One alignment begins with the first base of the read.
2. Both alignments together cover a number of bases in the read that is more than 2 bases longer than the length covered by any other single alignment.
3. Both alignments contain at least 5 read bases that do not overlap the other.
4. One alignment contains at least 10 read bases that do not overlap the other.
5. There are at most 20 bp unique to the read between matches to the reference.

If a pair of alignments passes this test, **breseq** generates the putative sequence of the new junction from the reference sequence and any intervening base pairs that are unique to the read.

In cases where the two read alignments overlap because some of its sequence could be assigned to match either location in the reference genome, **breseq** first trims each alignment to remove portions with mismatched bases or indels, then assigns as much of this overlap as possible to the side of the read that maps uniquely to the reference genome, or to the side with the lowest reference coordinate. It considers a location in the reference genome non-unique if it has repeat matches. If repeat\_region annotation exists in the reference genome, then **breseq** prefers to have junctions exactly overlap their boundaries.

This candidate junction sequence includes as many flanking reference bases on each end as the longest read in the entire data set. (So, if the data set consists of 36-bp reads, and the two read alignments overlapped by five bases, the sequence for this candidate junction would be 36 x 2 – 5 = 67 bases.

After processing every read in this manner, **breseq** combines all candidate junctions that match the same reference sequence and calculates a “position-hash” score. This score is a count of the number of different start positions in the reference sequence that are observed among the reads that support a candidate junction. This scoring scheme favors junctions supported by reads that are evenly distributed on each strand of the reference genome and evenly distributed at different positions relative to the junction point. (Pathological junction candidates tend to be supported only by reads that barely overlap the junction and are all on one strand.)

**breseq** sorts all candidate junctions according to this position-hash score, breaking ties with a “minimum-overlap” score. This score simply sums, over all reads, the minimum overlap that each read supporting the junction has to either of the two sides of the junction (not counting overlap regions). Top-scoring candidate junctions according to this two-tiered sorting scheme are retained until adding new candidates would cause their cumulative length to exceed 0.1x the total reference sequence length or their total number to exceed 5000.

### Scoring and accepting junctions¶

New junctions may also be supported by reads that do not overlap both sides sufficiently to seed alignments during mapping. To include these, **breseq** performs a second alignment step where it maps all reads to the new candidate junction sequences. Then, for each read, it determines whether its best alignment is to a junction candidate or to the reference sequence. For this purpose, alignments are assigned a score that is the number of matched reference bases minus the number of indel positions. Alignments that do not cover at least 28 bases of the read are discarded. Ties are resolved later.

A position-hash score is calculated again for each candidate junction by counting the number of different start positions that are observed among the reads that map best to that candidate junction. Junctions are tested in order from those with the most best alignments to those with the least or none. Reads that map equally well to the reference and to one or several junctions are included when calculating these position-hash scores.

Candidate junctions are accepted as evidence for a mutational event if their position-hash score exceeds a specified cutoff. This threshold is calculated by fitting a censored negative binomial (overdispersed Poisson) distribution to the read-depth coverage at unique-only reference positions as described under *Read coverage distribution*. **breseq** then calculates the chance that at least one read will start at any given position, on a given strand, according to this model. The chance of observing a given position-hash score is then calculated according to the binomial distribution assuming twice the read length number of trials (for each strand), and this chance per trial of observing a read in this register. A tail probability of 0.01 is used to establish the cutoff for this test.

In addition to this score, several other criteria are used when deciding whether a predicted junction has sufficient support. The complete list is:

1. Must have a pos-hash score greater than the calculated cutoff (as described above).
2. Must be supported by reads mapping to both strands of the predicted junction.
3. Must have reads mapping to the predicted junction that extend at least 14 base pairs into each side of the reference.
4. Must have reads mapping to each strand of the predicted junction that extend into each side of the junction at least 9 bp.
5. Must have reads mapping to the predicted junction where the side of the read with the smallest reference overlap extends at least 3 bp into the reference sequence on each side.

If the junction meets all of these criteria, it will be reported as evidence. In this case, reads that map equally elsewhere (to the reference or a different junction) are assigned to this junction and removed from further consideration. If, after all junction candidates have been tested, a read remains unused, it is assigned to the reference genome.

For junctions that pass this scoring cutoff, the ends of reads aligning to the junction are re-added as split sub-alignments to the alignment database, resolving ambiguously aligned bases, so that each read base is aligned to only one reference base. These split reads can be recognized in the output because they are renamed with suffixes of -M1 and -M2 for the two portions.

## Read alignment evidence (RA)¶

**breseq** calls base substitution mutations and small indels by examining each position in the pileup of mapped reads to the reference genome.

### Read end trimming¶

The ends of alignments of short reads to a reference sequence can be ambiguous with respect to insertion and deletion mutations. **breseq** uses a conservative strategy to ignore these bases when calling mutations.

**breseq** examines the reference sequence for perfect sequence repeats with lengths of 1-18 bases. Then for each position in the reference it determines how many bases must be trimmed from the end of a read beginning or ending at that position until the remaining bases are unambiguously aligned with respect to possible mutations causing changes in sequence repeats of these lengths. The minimum number of bases trimmed at each end of any read is 1, because one can never unambiguously know if another copy of that base was inserted by a mutation.

**Example of alignment end trimming.**

This example shows the number of bases that will be trimmed from the left and right ends of a read if its match to the reference genome begins or ends on that base. (Note that the strand of the genome that the read matches makes no difference!) The green, blue, and yellow highlight the repeats where the numbers come from for three test cases.

For green, a read with its left end aligned to this position is not informative with respect to how many AG copies there are in the sequenced genome. Therefore, it is only unambiguously aligned at the bases starting CAT-, and the first four bases will be trimmed. Similarly, a read with its right end aligned to the green position cannot tell how many TA copies there are. It will only be unambiguously aligned through -CTT, and its last four bases will be trimmed.

Trimming ends in this way enables more accurate mutation predictions because reads extending into these repeats from either side, but not completely crossing them, could otherwise be misinterpreted as evidence *against* a mutation.

For example, consider this mutation, which involves insertion of a new AGC at a site where there are already two AGC copies:

**Indel mutation prediction aided by end trimming.**

This image shows reads 1-6 aligned to the reference genome with and without end trimming (lowercase letters in reads). Two reads cross the entire AGCx2 repeat and show that a third AGC has been inserted.

Without end trimming, two reads on the top strand that do not cross the new AGC insertion, contradict that there was any change to the sequence here when they are aligned to the reference. With end trimming, these bases are ignored because they are ambiguous with respect to possible insertions, like the event that happened, or deletion of one AGC copy.

### Base quality re-calibration¶

In the FASTQ input files, each read base has generally been assigned a quality score by the normal pipeline for a given sequencing technology. Base quality re-calibration using covariates such as identity of the reference base, identity of the mismatch base, base position within the read, and neighboring base identities can significantly improve these error rate estimates [McKenna2010].

**breseq** uses an empirical error model that is trained by assuming that nearly all of the disagreements between mapped reads and the reference genome are due to sequencing errors and not bona fide differences between the sample and the reference: it simply counts the number of times that each base or a single-base gap is observed in a read opposite each base or a single-base gap. These counts are further binned by the quality score of the read base. (The quality score of the next aligned base in the read is used for single-base deletions). A pseudocount of one is added to counts in all categories, and these error counts are converted to error rates by dividing the count in each cell by the sum across that base quality score.

**Example of re-calibrated error rates.**

This plot shows a typical empirical error model fit to Illumina Genome Analyzer data. Notice that the rate of single-base deletions is much lower than the rate of any base miscall. Base qualities normally do not give information about the rates of indel mutations, and this re-calibration step allows **breseq** to estimate the rates of these sequencing errors.

Recall that **breseq** requires input in Sanger FASTQ format. Therefore the expected total error rate (E) at a given quality score (Q) before re-calibration is:

### Haploid Bayesian SNP caller¶

At each alignment position, **breseq** calculates the Bayesian posterior probability of possible sample bases given the observed read bases. Specifically, it uses a haploid model with five possible base states (A, T, C, G, and a gap), assumes a uniform prior probability of each state, and uses the empirical error model derived during base quality re-calibration to update the prior with each read base observation.

Thus, at a given alignment position, the log10 ratio of the posterior probability that the sample has a certain base b_x_ versus the probability that the sample has a different base is:

Where there are n reads aligned to this position, b_i_ is the base observed in the ith read, q_i_ is the quality of this base, and E is the probability of observing this read base given its quality score at a reference position with base b_x_ according to the empirical error model.

**breseq** determines the base with the highest value of L, and records read alignment evidence if this base is different from the reference base. This evidence is assigned log10 L minus the log10 of the cumulative length of all reference sequences as a new quality score for this base prediction.

Recall that **breseq** will only find indels of at most 2 bases as read alignment evidence, because all alignments with longer indels were split in a pre-processing step when predicting *New junction evidence (JC)*.

### Polymorphism Prediction¶

When **breseq** is called with the *breseq --polymorphism-prediction* option, it attempts to predict the frequencies of base substitution and short indel mutations that are present in only a fraction of the population (variants present in only a sub-population of individuals).

Warning

Polymorphism prediction is an experimental feature. While it has proven useful for some studies [Barrick2009b], it has not been thoroughly tested, has some known shortcomings (see below), and continues to be actively developed.

The prediction procedure uses the same empirical error model described above in *Base quality re-calibration*. This error model does not capture some second-order sources of variation in error rates that can lead to substantial numbers of false-positive polymorphism predictions. For example: sequencing error hotspots at certain positions, in certain contexts, and on certain strands. **breseq** also does not take into account read mapping qualities when weighing support for polymorphisms, which can lead to overconfidence in bases that result from misalignments.

Several additional **breseq** command-line options control steps that allow filtering out polymorphism predictions that pass the general statistical test for significance but display red-flag qualities indicative of biases. Top-scoring, but filtered, predictions are still displayed as “marginal predictions” in the **breseq** output, so that they can be manually examined.

The full list of polymorphism prediction options:

--polymorphism-prediction¶
:   Enable polymorphism prediction. Default: OFF.

--polymorphism-log10-e-value-cutoff <float>¶
:   Negative log10 of the E-value cutoff for the likelihood ratio test used in polymorphism prediction as described below. Default: 2. (Meaning the E-value must be ≤ 0.01 for a prediction to pass.)

--polymorphism-coverage-both-strands <int>¶
:   Minimum coverage (in number of uniquely mapped reads) required on each strand of the reference genome for both the major and the minor mutational variants to predict a polymorphism. Default: 0.

--polymorphism-frequency-cutoff <double>¶
:   Minimum frequency for the minor variant to predict a polymorphism. Default: 0.

--polymorphism-bias-p-value-cutoff <double>¶
:   P-value cutoff employed for both strand and quality score tests described below. Default: 0.05.

--polymorphism-reject-homopolymer-length <int>¶
:   Ignore polymorphisms predicted at reference genome positions in homopolymer repeats of this length or greater. Default: OFF.

--max-rejected-polymorphisms-to-show <int>¶
:   Show this many of the top polymorphism predictions that pass the likelihood-ratio test for significance but fail a bias filtering step. Default: 20.

#### Likelihood-ratio test for polymorphism¶

When polymorphism prediction is enabled **breseq** tests the hypothesis that reads aligned to each reference position (and base insertions relative to the reference) support a model that is a mixture of a major and minor mutational variant as opposed to a model that all disagreements with the reference sequence (or consensus change predicted as above) are due to sequencing errors. To do this, it calculates the chances of generating the observed alignment given the hypotheses that the sequenced sample consists of 100% of each of the four bases or a gap character and the per-base error model described above. Then it takes the base states giving the top and second highest probabilites and tests a mixture model that allows the major and minor variants to be present at any intermediate fraction in the sequenced population. **breseq** calculates the maximum likelihood percentage of each base state according to this model (at 0.1% resolution) and the chance of the data given this inferred reference distribution.

**breseq** then tests the statistical support for the model having only one reference base in the sequenced sample versus the model with one additional free parameter consisting of the ML mixture of two reference bases using a likelihood-ratio test. That is, twice the natural logarithm of the probability of the mixture model divided by the probability of the one-base model is compared to a chi-squared distribution with 1 degree of freedom.

As for the case of consensus mutation prediction from read alignment evidence, the p-value significance of the likelihood-ratio test is converted to an E-value by multiplying by the total number of reference positions.

#### Strand bias¶

This bias test uses Fisher’s Exact Test to calculate a two-sided p-value for the hypothesis that the top/bottom strand distribution of reads supporting the major base is not different from the top/bottom strand distribution of reads supporting the minor base. If the hypothesis is rejected when the p-value is compared to *--polymorphism-bias-p-value-cutoff*, then this may indicate that there was a sequencing-error hotspot in reads on one strand that generated a false-positive polymorphism prediction. This type of error happens frequently in data we have examined.

In practice, most problem predictions of this kind have zero or only a handful of reads on one strand and many reads on the other strand supporting the minor variant. This test can fail to reject false-positive predictions when there is low enough coverage of the minor variant that the test is not significant even if all of the reads supporting it are on one strand. In practice, this situation may be better dealt with by requiring there to be at least one read supporting the minor variant on each strand with *--polymorphism-coverage-both-strands*.

Conversely, if coverage is high there may be so many observations that a statistically significant bias is detected simply because library prep and sequencing is slightly more efficient on one strand due to the different sequence context, even when there is high coverage of all strand/base combinations. Use this option with caution in cases of very high coverage (>1000 reads).

#### Quality score bias¶

This bias test uses a one-sided Kolmogorov-Smirnov test to test whether base quality scores supporting the minor mutational variants are suspiciously lower than the base quality scores supporting the major variant. The p-value significance of rejecting the null hypothesis by this test is also compared to *--polymorphism-bias-p-value-cutoff*.

#### Homopolymer stretches¶

Currently, application of the error model in **breseq** on a per-column basis causes overprediction of indel polymorphisms in homopolymer stretches. Why is this the case? If there are 10 A’s in a row in the reference genome, deleting any one A will cause what looks like the same mutation after the gap is aligned to the rightmost reference position possible. Therefore, the actual chance of observing a deleted A is ten times the value expected from the error model. This discrepancy can make a small number of reads aligned to this position with deletions achieve significance by the likelihood-ratio test. Similar logic applies in the case of base insertions.

Currently, **breseq** cannot correct for these types of errors. In the interim, they can be filtered from the output by specifying the *--polymorphism-reject-homopolymer-length* option. A value of 5 gives reasonable results for *E. coli*. Generally, these false predictions also have extremely low frequencies (<2%) for the minor indel variants.

## Unknown base evidence (UN)¶

When there is insufficient evidence to call a base at a reference position, **breseq** reports this base as “unknown”. Contiguous stretches of unknown bases are output and shown in the results. Explicitly marking bases as unknown is useful when analyzing many similar genomes; it allows one to ascertain when a mutation found in certain data sets may have been missed in others due to low coverage and/or poor data quality in a particular sample.

## Missing coverage evidence (MC)¶

As **breseq** traverses read pileups it predicts deletions when it encounters reference regions with missing and low coverage.

### Read coverage distribution¶

If read sequences were randomly distributed across the entire reference sequence, then the number of positions with a given depth of read coverage would follow a Poisson distribution. In practice, the actual read coverage depth distribution deviates from this idealized expectation in at least two ways:

First, it is generally overdispersed relative to a Poisson distribution, e.g., there are more positions with higher and lower coverage than expected. This may represent a bias in the steps used to prepare a DNA fragment library or sequencing differences that cause more reads originating in certain regions of the genome to fail quality filtering steps. This overdispersion occurs even when re-sequencing a known genome. In fact, there is often a fingerprint of coverage bias where specific stretches consistently have higher or lower coverage than average across different instrument runs and DNA sample preps.

Second, there may be real mutations in the sample that affect the observed coverage distribution, such as large deletions and duplications. Deletions will add weight to the low end of the distribution because they cause reference positions to have zero or very low coverage. Non-zero coverage in true deletions is sometimes present in practice because there may be a small amount of contaminating DNA from a different sample that does not have this deletion or high error rate reads may spuriously map there. Duplications and amplifications will add weight to the distribution at higher coverage values.

**breseq** fits a negative binomial distribution (an overdispersed Poisson distribution) to the read coverage depth observed at unique-only reference positions. It uses left censored data to mitigate the effects of deleted regions on the overall fit. The threshold for censoring is determined by first finding the read depth with the maximum representaton in the distribution after smoothing using a moving average window size of 5 bases. Positions with coverage less than half this maximal read depth are ignored during fitting.

**Example of coverage distributon fit.**

In this example of real data, circles represent the number of positions in the reference with a given depth of read coverage. Data points that were censored during fitting are shown in red. The solid line is the least-squares best fit of a negative binomial distribution, and the dashed line is the best Poisson fit.

### Seed and extend algorithm¶

From the fit coverage distribution, **breseq** calibrates how it will call deletions. Deletion predictions are initiated at every reference position with unique-only coverage of zero. They are extended in each direction and merged until unique coverage exceeds a threshold calculated from the overall coverage distribution for the reference sequence. This cutoff is the the minimum threshold coverage *t* that satisfies the following relationship:

,

where *F* is the negative binomial cumulative distribution function with best-fit mean and size parameters and *L* is the reference sequence length.

In some cases there is ambiguity concerning the size of missing coverage regions because they encompass or overlap regions with repeat matches. Even if a specific example of a repetitive region is deleted, there will still appear to be coverage there because exact copies still exist elsewhere in the genome.

**breseq** assumes that any regions with repeat coverage that occur wholly within a region of low unique coverage (defined as above) have been deleted along with those flanking sequences. If a region of repeat coverage overlaps one end of the missing region prediction, then that end is assigned a range of possible reference positions. They reflect the two extreme possibilities that (1) the entire contiguous repetitive region is missing and (2) the entire contiguous repetitive region is still there. To determine the latter boundary, the same algorithm applied to unique coverage is used on unique coverage plus normalized repeat coverage depth, where normalization means that a repeat match counts as coverage of one divided by the total number of locations in the reference sequence that it matches.

**Coverage in a deleted reference region.**

This example shows a region of missing coverage (white background) that extends into a region of repeat coverage (red line), making the left side end of the missing coverage ambiguous.

## Mutation prediction¶

The previous sections describe **evidence** for mutations. **breseq** next tries to predict biologicaly relevant **mutational events** from this evidence. These rules are summarized in each section using *gdtools Utility Program* abbreviations for types of mutations and evidence.

### Base substitutions¶

*RA evidence = SNP or SUB mutation*

When the quality score of RA evidence is greater than a specified cutoff (the default is 6), a base substitution mutation is called. When only a single base is affected, **breseq** calls a base substitution (SNP) mutation. When multiple base substitutions occur adjacent to each other or in conjunction with indels (see below), **breseq** calls a substitution (SUB) mutation.

### Short insertions and deletions¶

*RA or JC evidence = INS, DEL, or SUB mutation*

For single-base insertions and deletions, RA evidence with gap characters is used to call mutations as in the case of base substitutions. For longer insertions and deletions, for which missing coverage evidence may not exist, these events may be predicted solely on the basis of new junctions joining them.

### Large deletions¶

*MC+JC evidence = DEL mutation*

Missing coverage typically indicates a large deletion event. When a junction also exists that precisely joins compatible endpoints, **breseq** predicts a deletion (DEL) mutation.

### Mobile element insertions¶

*JC+JC evidence = MOB mutation*

When two junctions exist that would join positions close by in the reference sequence to the ends of an annotated repeat\_region, **breseq** predicts a mobile element insertion (MOB). It further tries to shift the ends of the junctions such that they align best with the ends of the mobile element.

### Duplications¶

*JC evidence = AMP mutation*

If new junction evidence connects a region of the genome to a region upstream on the same strand, then it typically indicates that the intervening bases have been duplicated and **breseq** predicts a duplication. **breseq** currently does not use evidence from changes in read coverage depth to predict copy number, so coverage should be manually examined to verify this class of mutations.

### Other evidence¶

“Orphan” evidence that passed scoring thresholds but is not assigned by **breseq** to any of the mutational events above is shown in a separate section of the output so that it can be manually examined. **breseq** also displays some “marginal” evidence that fails the established cutoffs, but stil has some support, on a separate results page.

## Limitations¶

Even given perfect data, **breseq** cannot find some types of mutations:

Novel sequences, not existing in the reference
:   Because **breseq** maps reads to reference sequences, it will not find entirely novel sequences that have been inserted into the genome or novel extrachromosomal DNA fragments such as plasmids. Reads that do not map to the reference genome are dumped to an output file suitable for de novo assembly, so that they can be examined with other software programs.

Mutations in repeat regions
:   In genomic regions where the only mapped reads also match equally well to other locations in the genome, it is not possible to call mutations. This is an inherent limitation of short-read data. These regions are reported as ‘UN’ evidence, so that the user can distinguish where in the genome there was not sufficient coverage of uniquely mapped reads to call mutations.

Chromosomal inversions and rearrangements through repeat sequences
:   These types of mutations cannot be detected when they involve sequence repeats on the order of the read length. Reads that span repeats and uniquely align in the reference sequence on each end are necessary. **breseq** currently does not take advantage of mate-paired or paired-end information.

### Table Of Contents

- Methods
  - Read Mapping
  - New junction evidence (JC)
    - Identifying candidate junctions
    - Scoring and accepting junctions
  - Read alignment evidence (RA)
    - Read end trimming
    - Base quality re-calibration
    - Haploid Bayesian SNP caller
    - Polymorphism Prediction
      - Likelihood-ratio test for polymorphism
      - Strand bias
      - Quality score bias
      - Homopolymer stretches
  - Unknown base evidence (UN)
  - Missing coverage evidence (MC)
    - Read coverage distribution
    - Seed and extend algorithm
  - Mutation prediction
    - Base substitutions
    - Short insertions and deletions
    - Large deletions
    - Mobile element insertions
    - Duplications
    - Other evidence
  - Limitations

#### Previous topic

Test Drive

#### Next topic

Output

### This Page

- Show Source

### Quick search


Enter search terms or a module, class or function name.

### Navigation

- index
- next |
- previous |
- breseq 0.25 documentation »

© Copyright 2014 Jeffrey E. Barrick and contributors.
Created using Sphinx 1.1.
