# Supplementary material for: Identifying structural variation in haploid microbial genomes from short-read resequencing data using breseq
Source: BMC Genomics. 2014 Nov 29;15(1):1039. doi: 10.1186/1471-2164-15-1039 (PMC4300727; doi:10.1186/1471-2164-15-1039)
Supplement: Supplementary file 1 — Additional file 1: [breseq-0.25.tar.gz]. Source code archive for breseq computational pipeline (version 0.25). (ZIP 9 MB) [file 12864_2014_6751_MOESM1_ESM.zip › breseq-0.25.tar.gz!/breseq-0.25/documentation/output.html]

Output — breseq 0.25 documentation


### Navigation

- index
- next |
- previous |
- breseq 0.25 documentation »

# Output¶

## HTML Archive¶

**breseq** produces results as a stand-alone HTML archive in the output directory. You can load these files directly in a browser, or copy the directory to a server to allow access via the web.

Important files include:

output/index.html
:   The main results page. It consists of an upper table showing predicted mutational events and possibly several other tables showing high-quality “orphan” evidence that **breseq** was unable to assign to mutational events. The format of each row varies depending on the type of mutation, as described in *Mutation Display* and *Evidence Display*.

output/marginal.html
:   Result page showing evidence for mutations with marginal support. Specifically: (1) RA evidence that supports a mutated base or indel more than the reference sequence, but without sufficient support to pass the cutoff threshold, and (2) JC evidence for a set number of the highest scoring junctions that do not pass all test criteria. The format of these tables is described in *Evidence Display*.

output/summary.html
:   Additional information about the read files, reference sequences, analysis settings, and results. Links to plots showing the re-calibrated base error model, coverage distribution for each reference sequence, and coverage across each reference sequence.

output/output.gd
:   Text file of evidence and mutation predictions in computer-readable *gdtools Utility Program*. This file can be used as input to certain analysis programs to compare mutations found in different samples.

output/log.txt
:   The original command line used to invoke **breseq**.

### Mutation Display¶

Each row displays a predicted mutation in the re-sequenced sample relative to the reference. Examples showing how the format varies depending on the type of mutation are provided in the following sections.

Column descriptions:

evidence
:   Links to the types of evidence that support this particular prediction. See *Evidence Display*.

seq id
:   The identifier for the reference sequence with the mutation. This column will not be shown for mutations if there is only one reference sequence.

position
:   Position in the reference sequence of the mutation. Generally this is where the mutation begins when the mutation affects a range of positions.

mutation
:   Description of the mutation. Typically describes how nucleotides are added, substituted, or deleted. May also refer to a mobile element in the genome and how it is inserted at the specified position.

annotation
:   Description of the mutation. For base substitutions inside genes, shows the resulting amino acid and codon changes. For other mutations inside genes, gives the local coordinates of the nucleotides affected. For mutations in intergenic regions, gives two relative positions (e.g., +150/-119) where the numbers are the distances from the mutation to the nearest neighboring genes before and after it in the genome, and the +/- signs indicate whether the mutation is oriented upstream or downstream with respect to each of these genes.

gene
:   Gene or genes affected by the mutation. May be a single gene, an intergenic region (gene1/gene2), a range that covers several genes (gene1–geneN). Brackets around a [gene] in a range mean that the mutation begins or ends within that gene.

description
:   Descriptions of the genes affected by the mutation. Generally these correspond to the genes in the gene column, but if many genes are affected, this field is abbreviated to a list of all affected gene names.

All gene information is taken from input GenBank files. How informative descriptions are about the effects of mutations is entirely dependent on the quality of annotation in the reference sequence files.

#### Single-base substitution (SNP)¶

Replacement of the reference T at position 70,867 with a C inside the *araA* gene. This mutation changes the 92nd codon of *araA* from GAC to GGC, causing an aspartic acid (D) to glycine (G) substitution in the encoded protein. The base change in the codon is the reverse-complement of the base change in the genome because this gene is encoded on the bottom strand of the reference sequence.

Replacement of the reference T at position 1,298,712 with a G in the intergenic region between the *ychE* and *oppA* genes. The mutation is downstream of *ychE* by 674 bases (because this gene is before it in position and on the top strand of the reference) and upstream of *oppA* by 64 bases (because this gene is after it in position and also on the top strand of the genome).

#### Multiple-base substitution (SUB)¶

Replacement of two adjacent reference bases at positions 47,977 and 47,978 with AC in an intergenic region. This mutation is near the end of the genome, so there is no gene after it. It is downstream of *lambdap79* by 33 bases (because this gene is before it in position and on the top reference strand).

#### Insertion (INS)¶

For insertion mutations, new bases are added after the specified *position*.

Insertion of a G after reference position 3,893,551. This mutation is 6 nucleotides downstream of *kup* and 50 nucleotides upstream of *insJ-5*.

Insertion of CC after reference position 3,290,071 inside the *gltB* gene. This mutation occurs after the 205th base of the 4554-base open reading frame of this gene.

#### Deletion (DEL)¶

For deletion rows, the *position* column gives the first missing reference base and the *mutation* column gives the size of the deletion. Thus, the deleted reference region extends from *position* to *position* + *size* -1.

A 6,934-base deletion starting at position 3,894,997. The *annotation* column reports that it is IS150-mediated, because this repetitive element occurs on one margin of the deletion. This deletion begins before the *rbsD* gene and ends within the *yieO* gene. This mutation is supported by *New Junction (JC)* and *Missing coverage (MC)* evidence.

A single-base deletion at position 1,332,148 in an intergenic region. The deleted nucleotide is located 131 bp downstream of the *topA* gene and 79 bp upstream of the *cysB* gene. This mutation is supported by *Read alignment (RA)*.

#### Mobile element insertion (MOB)¶

Mobile element insertions can result in duplications of the target site. The provided *position* is the first of such possibly duplicated bases. The number of bases in parentheses in the *annotation*, e.g. (+7) bp, are duplicated, starting with the indicated *position*, so that they now occur before and after the new copy of the mobile element. Additional bases may be added or deleted as a result of the mobile element insertion at either end. These are indicated outside of double colons (::) on the affected side of the mobile element name in the *annotation* column. The strand of the newly inserted mobile element is indicated in parentheses after its name.

Insertion of an IS*3* element in the reverse orientation. Bases 3,571,196 through 3,571,198 are duplicated, so that they now occur on each margin of the newly inserted element. In addition, the sequence TCA was added directly after the IS*3* element on the right margin. The duplicated bases are positions 397 through 399 of the 435-base *uspA* reading frame.

Insertion of an IS*186* element in the forward orientation. Bases 4,524,522 through 4,524,527 are duplicated, so that they now occur on each margin of the newly inserted element. These bases are 494 through 499 of the 549-base *fimA* reading frame.

Insertion of an IS*186* element in the forward orientation. Bases 2,736,667 through 2,736,675 are duplicated, so that they now occur on each margin of the newly inserted element. Two bases of the mobile element on the left margin were lost, apparently during insertion. The duplicated bases are 818 through 826 of the 1425-base *ascB* reading frame.

#### Sequence amplification (AMP)¶

For duplications and other tandem amplifications, *position* indicates the first repeated base.

Duplication of 8 bp inside the *pykF* gene. The bases 1,733,290 to 1,733,297 now appear twice at this location. This mutation would cause a frameshift.

### Evidence Display¶

Evidence is shown in tables with different fields from mutation predictions, that provide more detailed information about support for genomic changes. Clicking on any evidence link for a mutation prediction will also bring up pages with tables showing all items of evidence that **breseq** used to predict the mutational event.

#### New Junction (JC)¶

Each JC row consists of two sub-rows, one describing one side of the junction in the reference sequence. If a sub-row is highlighted in orange, it means that side of the junction ambiguously maps to more than one place in the reference. In this case, the coordinate shown is an example of one site.

Column descriptions:

\* link
:   Links to a results page showing the sequence of the new junction as the reference and all reads aligned to the junction.

? links
:   Links to a results pages for each side of the juncton, that show the reference sequence at that site and any reads that aligned better to this original sequence than to the new junction. Note that in some cases (such as tandem duplications), it is possible for both the new and old junction sequences to exist in the sample. You can check for this by examining these read alignments. Sequences where the read name has a -M1 or -M2 appended are reads that mapped better to the new junction.

seq id
:   Identifiers for the reference sequences involved in the new junction.

position
:   Positions in the reference sequence of the two sides of the new junction. Each position has an equals sign (=) before or after it that represents how the junction was constructed. The reference sequence approaches that coordinate from the side with the equals sign. The displayed coordinates are juxtaposed with each other to make the new junction.

reads
:   The total number of reads that map to this junction.

score
:   The position-hash score for the junction in **<bold angle brackets>** and the minimum-overlap score on the next line.

annotation, gene, product
:   Description of the effects of this change on each side of the junction. The format of these columns is the same as in *Mutation Display*.

Examples:

This image shows the page from clicking on the \* link for this junction. A partial alignment of reads to the new junction is shown. Notice the two joined pieces of the reference sequence at the top that they align to. This sequence is on the bottom strand of the reference if start is greater than end.

This image shows the page from clicking on one of the ? links for this junction. Notice that only a piece of the reads maps to this region and that it ends where these reads begin matching a disjoint region in the reference genome. Clearly the old junction is not supported by any reads in this sample and must no longer exist. Once again, only a partial alignment is shown.

#### Read alignment (RA)¶

Column descriptions:

\* link
:   Links to a results page showing the alignment of reads to this position.

seq id
:   Identifier for the reference sequence where the change is located.

position
:   Position in the reference sequence of the single base substitution, insertion, or deletion. It consists of two parts. The first is the reference position, the second is an “insert count” that - if greater than zero - indicates this is in a column of the alignment that does not exist in the reference sequence (i.e., it is an insertion relative to the reference and is this many columns past the specified reference position.).

change
:   The base change, deletion, or insertion.

freq
:   Frequency of this base change in the sample. **breseq** currently only predicts mutations of 0% or 100% frequency.

score
:   The base-10 logarithm ratio of the posterior probability that this position in the sample is the called base to the probability that it is any other base, minus the base-10 logarithm of the total number of positions in all reference sequences. The higher the score, the more evidence for the mutation.

cov
:   The number of reads overlapping the mutation. Note that portions of reads that are not aligned (lowercase bases with a white background), ends of reads that have been trimmed because alignments their may be ambiguous (lowercase bases with a colored background), and read positions with very low base quality scores that typically indicate sequencing errors (highlighted in yellow) are not counted in this coverage number.

annotation, gene, product
:   Description of the change’s effects for each side of the junction. The format of these columns is the same as in *Mutation Display*.

Example:

Partial alignment of reads showing that most support a base substitution. The > and < for each named read indicate the strand of the reference sequence that it matched (top and bottom, respectively).

#### Missing coverage (MC)¶

Column descriptions:

\* links
:   Links to results pages showing the alignment of reads to the left and right margins of the region with missing coverage.

÷ link
:   Link to the results page showing a plot of the read coverage in the region of the msising coverage.

seq id
:   Identifier for the reference sequence where the change is located.

start, end, size
:   The start and end reference positions and size of the missing coverage. May indicate a range of positions when one end of the missing coverage is in a repeat region.

← cov
:   Unique read coverage depth on the left margin of the region of missing coverage. Coverage at the last position outside the region is shown followed by coverage at the first position inside the region of missing coverage in brackets.

→ cov
:   Unique read coverage depth on the right margin of the region of missing coverage. Coverage at the last position inside the region is shown followed by coverage at the first position outside the region.

gene, description
:   Description of the change’s effects for each side of the junction. The format of these columns is the same as in *Mutation Display*.

Example:

Read coverage depth around the missing coverage. The white area shows the maximal boundaries of the predicted range.

The graphed lines are labeled “unique” for reads with only one best match to the reference genome and “repeat” for multiple equally good matches to repeat sequences (which are down-weighted by how many matches they have, i.e. a read matching three places contributes 1/3 to the coverage depth at each matched site). Within each type coverage is graphed separately for reads mapping to the “top” and “bottom” strands of the reference sequence (i.e., forward and reverse complement matches) to aid in detecting artifacts, and these sum to the “total” coverage value.

## Processed Data¶

**breseq** outputs several files that can be used by other software programs to further analyze the final processed read data.

data/reference.bam, data/reference.bam.bai
:   The BAM (Binary SAM) formatted database of read alignments to the reference and its index. Along with the *reference.fasta\** files can be used with any **SAMtools** compatible program.

data/reference.fasta, data/reference.fasta.fai
:   File of all reference sequences and the corresponding index. Along with the *reference.fasta\** files can be used with any **SAMtools** compatible program.

data/<read\_file>.unmatched.fastq
:   These files contain reads from each original file that were not mapped to the reference sequences. This file can be used for de novo assembly to predict if there are novel sequences in the sample.

## Viewing Output / Aligned Reads in the IGV¶

You can visualize the “raw data” (how **breseq** aligned reads to the reference genome) using the Integrative Genomics Viewer (IGV) and files located in the data folder created by **breseq**.

1. Install and open IGV
2. Import the reference genome sequence:

> - Click ‘File’, and then ‘Import Genome...’
> - Fill out the requested information: ‘ID’, ‘Name’
> - Choose the FASTA file: data/reference.fasta.
> - The other fields are optional.

3. Import the reference genome feature information:

> - Click ‘File’, and then ‘Load from File...”
> - Choose the GFF3 file: data/reference.gff3.

4. Import the read alignments to the reference genome:

> - Click ‘File’, and then ‘Load from File...”
> - Choose the BAM file: data/reference.bam.

### Table Of Contents

- Output
  - HTML Archive
    - Mutation Display
      - Single-base substitution (SNP)
      - Multiple-base substitution (SUB)
      - Insertion (INS)
      - Deletion (DEL)
      - Mobile element insertion (MOB)
      - Sequence amplification (AMP)
    - Evidence Display
      - New Junction (JC)
      - Read alignment (RA)
      - Missing coverage (MC)
  - Processed Data
  - Viewing Output / Aligned Reads in the IGV

#### Previous topic

Methods

#### Next topic

References

### This Page

- Show Source

### Quick search


Enter search terms or a module, class or function name.

### Navigation

- index
- next |
- previous |
- breseq 0.25 documentation »

© Copyright 2014 Jeffrey E. Barrick and contributors.
Created using Sphinx 1.1.
