# Supplementary material for: Identifying structural variation in haploid microbial genomes from short-read resequencing data using breseq
Source: BMC Genomics. 2014 Nov 29;15(1):1039. doi: 10.1186/1471-2164-15-1039 (PMC4300727; doi:10.1186/1471-2164-15-1039)
Supplement: Supplementary file 1 — Additional file 1: [breseq-0.25.tar.gz]. Source code archive for breseq computational pipeline (version 0.25). (ZIP 9 MB) [file 12864_2014_6751_MOESM1_ESM.zip › breseq-0.25.tar.gz!/breseq-0.25/documentation/references.html]

References — breseq 0.25 documentation


### Navigation

- index
- next |
- previous |
- breseq 0.25 documentation »

# References¶

|  |  |
| --- | --- |
| [Barrick2009a] | Barrick et al. (2009) Genome evolution and adaptation in a long-term experiment with *Escherichia coli*. *Nature* **461**:1243-1247. |

|  |  |
| --- | --- |
| [Barrick2009b] | Barrick and Lenski. (2009) Genome-wide mutational diversity in an evolving population of *Escherichia coli*. *Cold Spring Harb. Symp. Quant. Biol.* **74**:119-129. |

|  |  |
| --- | --- |
| [McKenna2010] | McKenna et al. (2010) The Genome Analysis Toolkit: A MapReduce framework for analyzing next-generation DNA sequencing data. *Genome Research* **20**:1297-1303. |

#### Previous topic

Output

#### Next topic

Acknowledgments

### This Page

- Show Source

### Quick search


Enter search terms or a module, class or function name.

### Navigation

- index
- next |
- previous |
- breseq 0.25 documentation »

© Copyright 2014 Jeffrey E. Barrick and contributors.
Created using Sphinx 1.1.
