# Supplementary material for: Identifying structural variation in haploid microbial genomes from short-read resequencing data using breseq
Source: BMC Genomics. 2014 Nov 29;15(1):1039. doi: 10.1186/1471-2164-15-1039 (PMC4300727; doi:10.1186/1471-2164-15-1039)
Supplement: Supplementary file 1 — Additional file 1: [breseq-0.25.tar.gz]. Source code archive for breseq computational pipeline (version 0.25). (ZIP 9 MB) [file 12864_2014_6751_MOESM1_ESM.zip › breseq-0.25.tar.gz!/breseq-0.25/documentation/search.html]

Search — breseq 0.25 documentation


### Navigation

- index
- breseq 0.25 documentation »

# Search

Please activate JavaScript to enable the search
functionality.

From here you can search these documents. Enter your search
words into the box below and click "search". Note that the search
function will automatically search for all of the words. Pages
containing fewer words won't appear in the result list.

### Navigation

- index
- breseq 0.25 documentation »

© Copyright 2014 Jeffrey E. Barrick and contributors.
Created using Sphinx 1.1.
