# Supplementary material for: Identifying structural variation in haploid microbial genomes from short-read resequencing data using breseq
Source: BMC Genomics. 2014 Nov 29;15(1):1039. doi: 10.1186/1471-2164-15-1039 (PMC4300727; doi:10.1186/1471-2164-15-1039)
Supplement: Supplementary file 1 — Additional file 1: [breseq-0.25.tar.gz]. Source code archive for breseq computational pipeline (version 0.25). (ZIP 9 MB) [file 12864_2014_6751_MOESM1_ESM.zip › breseq-0.25.tar.gz!/breseq-0.25/documentation/test_drive.html]

Test Drive — breseq 0.25 documentation


### Navigation

- index
- next |
- previous |
- breseq 0.25 documentation »

# Test Drive¶

In this test drive, we will first download a bacterial genome and FASTQ files of Illumina reads. Then, we will use **breseq** to predict mutations that are present in the re-sequencing data relative to this reference genome.

## 1. Download data files¶

First, create a directory called **tutorial**.

### Reference sequence¶

**breseq** needs the reference sequence in Genbank format. In this example, the reference sequence is *Escherichia coli* B strain REL606. The Genbank (Refseq) accession number is: **NC\_012967** . You can search for this sequence at http://www.ncbi.nlm.nih.gov/ or follow this direct link.

Once the sequence is displayed, you will want to select “Show sequence” from the Display options on the right then click update view. Finally, use the send menu to choose “Complete Record” and Destination: “File” and “Genbank (Full)”. It should start downloading a file called “sequence.gb”. Rename this to **NC\_012967.gbk** after it downloads.

### Read files¶

We’re going to use Illumina genome re-sequencing data from a strain that evolved for 20,000 generations in a long-term evolution experiment [Barrick2009a]. This data is available in the European Nucleotide Archive (ENA). Go to http://www.ebi.ac.uk/ and search for the accession number: **SRR030257**. Then click on the accession number to open the record and download the two data files using the links in the ‘ftp’ column.

Move all three of these files into the **tutorial** directory that you created.

## 2. Run **breseq**¶

Check to be sure that you have changed into the **tutorial** directoryand that you have all of the input files (and have uncompressed them).

```
>>> ls
NC_012967.gbk           SRR030257_1.fastq       SRR030257_2.fastq
```

Now, run breseq:

```
>>> breseq -r NC_012967.gbk SRR030257_1.fastq SRR030257_2.fastq
```

The first named argument (-r) is the reference sequence. If you had multiple reference sequences, you could input multiple ones (e.g., -r NC\_012967.gbk -r plasmid.gbk).

The unnamed arguments at the end of the command line are the read files. You can input as many as you need and mix FASTQ files from different sequencing technologies (e.g., Illumina and 454).

Warning

Running **breseq** on a full data set like this is not fast. Depending on your computer, this could take several hours. To speed things up, you can set -j option to the number of cores on your machine to enable multithreaded execution of some steps (e.g., -j 4 for a quad-core machine). If you want to speed this example up, you might also include only one of the two input read files on the command line.

## 3. Open **breseq** output¶

Open the file **index.html** in the new **output** directory. This describes the predicted mutations and also evidence for mutations that **breseq** could not resolve into mutational events. The tables in this HTML file are more fully described in the section on *Output*.

### Table Of Contents

- Test Drive
  - 1. Download data files
    - Reference sequence
    - Read files
  - 2. Run **breseq**
  - 3. Open **breseq** output

#### Previous topic

Usage Summary

#### Next topic

Methods

### This Page

- Show Source

### Quick search


Enter search terms or a module, class or function name.

### Navigation

- index
- next |
- previous |
- breseq 0.25 documentation »

© Copyright 2014 Jeffrey E. Barrick and contributors.
Created using Sphinx 1.1.
