# Supplementary material for: Identifying structural variation in haploid microbial genomes from short-read resequencing data using breseq
Source: BMC Genomics. 2014 Nov 29;15(1):1039. doi: 10.1186/1471-2164-15-1039 (PMC4300727; doi:10.1186/1471-2164-15-1039)
Supplement: Supplementary file 1 — Additional file 1: [breseq-0.25.tar.gz]. Source code archive for breseq computational pipeline (version 0.25). (ZIP 9 MB) [file 12864_2014_6751_MOESM1_ESM.zip › breseq-0.25.tar.gz!/breseq-0.25/documentation/usage.html]

Usage Summary — breseq 0.25 documentation


### Navigation

- index
- next |
- previous |
- breseq 0.25 documentation »

# Usage Summary¶

## **breseq**¶

Usage:

```
breseq -r reference1.gbk [-r reference2.gbk ...] reads1.fastq [reads2.fastq, reads3.fastq...]
```

Run the **breseq** pipeline for mutation prediction from genome re-sequencing data.

Required options:

-r <file\_path>, --reference <file\_path>¶
:   Input reference genome sequence files in GenBank format. If there are multiple reference sequences stored in separate GenBank files (e.g., a bacterial genome and a plasmid), this option can be supplied multiple times.

reads1.fastq [reads2.fastq, reads3.fastq...]
:   The remaining arguments at the command line are the FASTQ input files of reads. FASTQ files with base quality scores that are not in SANGER format will be converted. In addition, reads with >50% N bases will be removed from the converted FASTQ file by default. **breseq** re-calibrates the error rates for each FASTQ file separately, so data sets that were generated independently should be stored in different input files.

Commonly used options:

> option:: -h, –help
>
> Produce help message showing advanced options.

-n <string>, --name <string>¶
:   Human-readable name of the analysis run for output (DEFAULT=<none>).

-j <int>, --num-processors <int>¶
:   Number of processors to use in multithreaded steps (DEFAULT=1).

--no-junction-prediction¶
:   Do not predict new sequence junctions.

-p, --predict-polymorphisms¶
:   Predict polymorphic (mixed) mutations.

For a complete list of options, please see the command line help.

## Command: bam2aln¶

Usage:

```
breseq BAM2ALN [-b reference.bam -f reference.fasta -o alignment.html -n 200] region1 [region2 region3 ...]
```

Display reads aligned to the specified region or regions.

Commonly used options:

-b <file\_path>, --bam <file\_path>¶
:   BAM database file of read alignments (DEFAULT=data/reference.bam).

-f <file\_path>, --fasta <file\_path>¶
:   FASTA file of reference sequences (DEFAULT=data/reference.fasta).

-o <path>, --output <path>¶
:   Output path. If there is just one region, the name of the output file (DEFAULT=region1.\*). If there are multiple regions, this argument must be a directory path, and all output files will be output here with names region1.\*, region2.\*, ... (DEFAULT=.).

-r <region> , --region <region> , --unnamed-arguments-- <region> [<region2>, <region2>, ...]¶
:   Regions to create alignments for. Must be provided as sequence regions in the format **ACCESSION:START-END**, where **ACCESSION** is a valid identifier for one of the sequences in the FASTA file, and **START** and **END** are 1-indexed coordinates of the beginning and end positions. Any read overlapping these positions will be shown. A separate output file is created for each region. Regions may be provided at the end of the command line as unnamed arguments.

-n <int>, --max-reads <int>¶
:   Maximum number of reads that will be aligned to a region. If there are more than this many reads, then the reads displayed are randomly chosen and a warning is added to the output. (DEFAULT=200).

## Command: bam2cov¶

Usage:

```
breseq BAM2COV [-b reference.bam -f reference.fasta --format PNG -o output.png] region1 [region2 region3 ...]
```

Create a coverage plot or table for the specified region or regions.

Commonly used options:

-b <file\_path>, --bam <file\_path>¶
:   BAM database file of read alignments (DEFAULT=data/reference.bam).

-f <file\_path>, --fasta <file\_path>¶
:   FASTA file of reference sequences (DEFAULT=data/reference.fasta).

-o <path>, --output <path>¶
:   Output path. If there is just one region, the name of the output file (DEFAULT=region1.\*). If there are multiple regions, this argument must be a directory path, and all output files will be output here with names region1.\*, region2.\*, ... (DEFAULT=.).

-r <region>, --region <region>, --unnamed-arguments-- <region> [<region2>, <region2>, ...]¶
:   Regions to create alignments for. Must be provided as sequence regions in the format **ACCESSION:START-END**, where **ACCESSION** is a valid identifier for one of the sequences in the FASTA file, and **START** and **END** are 1-indexed coordinates of the beginning and end positions. Any read overlapping these positions will be shown. A separate output file is created for each region. Regions may be provided at the end of the command line as unnamed arguments.

--format <PNG/PDF>¶
:   Format of output plot: PNG or PDF. (DEFAULT=PNG).

-t, --table¶
:   Create tab-delimited file of coverage instead of a plot.

-1, --total-only¶
:   Only plot/tabulate the total coverage at a position. That is, do not not output the coverage on each genomic strand.

--resolution <int>¶
:   Number of positions to output coverage information for in interval (0=ALL) (DEFAULT=600).

### Table Of Contents

- Usage Summary
  - **breseq**
  - Command: bam2aln
  - Command: bam2cov

#### Previous topic

Installation

#### Next topic

Test Drive

### This Page

- Show Source

### Quick search


Enter search terms or a module, class or function name.

### Navigation

- index
- next |
- previous |
- breseq 0.25 documentation »

© Copyright 2014 Jeffrey E. Barrick and contributors.
Created using Sphinx 1.1.
